# Inflexible neurobiological signatures precede atypical development in infants at high risk for autism

**DOI:** 10.1038/s41598-017-09028-0

**Published:** 2017-09-12

**Authors:** Kristina Denisova, Guihu Zhao

**Affiliations:** 1 Sackler Institute for Developmental Psychobiology, Columbia University College of Physicians and Surgeons, New York, NY 10032 USA; 2 Department of Psychiatry, Columbia University College of Physicians and Surgeons, New York, NY 10032 USA; 3 Division of Developmental Neuroscience, New York State Psychiatric Institute, New York, NY 10032 USA

## Abstract

Variability in neurobiological signatures is ubiquitous in early life but the link to adverse developmental milestones in humans is unknown. We examined how levels of signal and noise in movement signatures during the 1st year of life constrain early development in 71 healthy typically developing infants, either at High or Low familial Risk (HR or LR, respectively) for developing Autism Spectrum Disorders (ASD). Delays in early learning developmental trajectories in HR infants (validated in an analysis of 1,445 infants from representative inf﻿ant-sibling studies) were predicted by worse stochastic patterns in their spontaneous head movements as early as 1–2 months after birth, relative to HR infants who showed more rapid developmental progress, as well as relative to all LR infants. While LR 1–2 mo-old infants’ movements were significantly different during a language listening task compared to during sleep, HR infants’ movements were more similar during both conditions, a striking lack of diversity that reveals context-inflexible experience of ambient information. Contrary to expectation, it is not the level of variability *per se* that is particularly detrimental in early life. Rather, inflexible sensorimotor systems and/or atypical transition between behavioral states may interfere with the establishment of capacity to extract structure and important cues from sensory input at birth, preceding and contributing to an atypical brain developmental trajectory in toddlerhood.

## Introduction

Humans begin life as fussy and curious infants, normally rapidly and effortlessly establishing an ability to exploit statistical regularities in the unstructured acoustic, visual, and sensory stream during the first year of life. Drawing on a combination of powerful innate and experiential mechanisms, this critical redundancy-reducing mechanism in adulthood helps guide future inference when encountering new information across different domains, including rule abstraction for efficient category parsing^1^, sensitivity to biomechanical plausibility in visual representation of shape^2^, the use of object features for efficient compression into memory^3^ and learning language^4^.

A fundamental problem in developmental science is to understand how early perturbations in the nature of experiencing this ambient information may contribute to an atypically developing mind and brain. Part of the problem is that measures of implicit function are challenging to obtain in very young human infants. Infant studies relying on variants of the preferential looking method (e.g., ref. 5) provide critical insights into infant competencies, but often experience substantial attrition rates (due to infants’ “fussiness”; with rates as high as 50%: e.g., ref. 6). The Blood Oxygenation-Level-Dependent (BOLD) signal collected during a resting-state or functional Magnetic Resonance Imaging (MRI) scan allows *in-vivo*, non-invasive study of brain function but is rendered unreliable if a participant moves excessively during the scan. (Additional technological challenges in the scanning of neonates exist, including but not limited to, low contrast between gray and white matter due to weak myelination; this makes it challenging to distinguish gray and white matter precisely. With regard to the “preferential looking” studies, there are also important neuroanatomical maturational constraints, with vision being guided initially by the retinocollicular system, with control shifted to cortical areas (V1) of the visual system around 3 months; this means that it is challenging to study certain (spatial vs. temporal) attributes of visual information in very young infants).

Variability in natural physiological movement signals is thus ubiquitous in early life, a fact that we harness here to probe early features of atypical development. A second piece of the puzzle is that neonates have extremely unique, substantial requirements for rest (or sleep) during the first few months of life relative to adult humans, spending the majority of their time sleeping (up to 70%, ~17 out of 24 hours^7, 8^) in order to develop proper sensorimotor maps of their world^9, 10^. Surprisingly, no previous work has compared potential perturbations in natural, spontaneous movements during sleep relative to those during wakefulness, such as when the infant is listening to human speech.

In the fraction of time that the infant spends awake, he or she is likely to be exposed to a variety of stimuli, gradually developing attunement both to the structure of inputs available in the environment and to the self-constraints that may limit performance. For instance, visual statistical learning experiments show that typically developing (TD) 2-, 5-, and 8-mo old infants are sensitive to violations of transitional probabilities that define pairs of visual attributes in sequences, thereby demonstrating an “early learning mechanism’’ that detects structure in the environment^11^. Additionally, twelve to thirteen month olds use temporal regularities (organizing information via chunking) to boost their total memory capacity^12^, indicating that working memory serves as an important constraint under which the naïve learner operates while attending to potential structure available in the environment. When inferring whether a toy failure is due to their actions or to the toy itself, 16-mo old infants rely on minimal data to track “statistical dependence between agents, objects, and outcomes” in order to infer the most “rational” choice or action^13^. These studies highlight the importance of active exploration and interaction of the infant with the surrounding environment early in life.

Infants typically use information available in their ambient auditory stream to gradually master the phonology and grammar of their native language^14^. For example, TD 9-mo infants show a sensitivity to ‘legal’ (permitted) vs. ‘illegal’ native-language phonotactic (syllable clustering) combinations^15^ demonstrating an early capacity for distributional learning (e.g., refs 16 and 17), an ability that is not limited to processing natural languages^18^. As evidence of developmental progression of this ability, TD infants show increasing improvement or “tighter clustering” in vowel categories at 16 (vs. 12) weeks and again at 20 (vs. 16) weeks^19^, suggesting that “linguistic experience alters speech perception” in the first 6 months of life. Aslin & Newport (2012) note that the “consistency of how the context cues are distributed across strings of input determines whether a rule is formed… or whether specific instances are learned”^4, 20^, suggesting a common learning mechanism that underlies both statistical learning and rule-governed aspects of language development. Further, at least some aspects of this language learning mechanism (especially those that support statistic extraction and implicit decision-making^4^) are generalizable to the learning of information in a different domain^4^ as well (cf. ref. 11).

Crucially for our study, neurobiological evidence in human adults and infants indicates a robust connection between motor and perceptual mechanisms that resemble an “action-perception link”^14, 21^ or a “perceptual-motor link”^22^. For example, a functional MRI study in adults (~27 year olds) found activation of the same motor circuits during a listening task as those that participate during articulatory motor processes^21^. Further, the role of language-specific motor circuits is updated as a function of experience with auditory input. Kuhl and colleagues^14^ studied language perception in a native- vs. non-native language context and found that 7-mo olds activate both motor and language (superior temporal) areas similarly to native- and non-native sounds, whereas 11–12-mo olds show greater activation of motor brain areas to non-native relative to native speech^14^. Taken together, these findings suggest that the (*i*) motor system contributes to an early learning mechanism, and (*ii*) this mechanism undergoes continued tuning in the developing brain.

It is not known how variability in the structure of experiencing sensorimotor information in early life contributes to an atypical or delayed development measured later, around 6, 12, and/or 18 months. In the context of atypical development, recent fMRI work has shown that children and adults with Autism Spectrum Disorders (ASD) who have higher noise levels in spontaneous movements also show increased behavioral task-related uncertainty relative to TD participants; further, individuals with ASD who show higher (vs. lower) levels of uncertainty in behavior show atypically increased functional connectivity between the bilateral insula and the pre-frontal cortex^23^. This work raises the possibility that (*iii*), atypical maturation of motor mechanisms in early life may affect the integrity of developmental trajectories in infants at High vs. Low familial Risk for developing ASD.

We examine how the balance of signal and noise in fluctuations of head movements during the two principal states of being—sleep and wakefulness, while listening to human language—in the first year of life may predict general developmental trajectories in 71 human infants, either at High or Low familial Risk for ASD (“HR” infants are considered those with a biological sibling diagnosed with ASD). Infants underwent two different scans: a resting-state functional MRI during which infants were naturally sleeping, which is the focus of the current work, as well as an fMRI during which infants listened to native language. Using a technique we term “upcycling” for neuroimaging, we first (*i*) use established image-based methods to estimate volume-by-volume subject movement during the scan^24^ and then (*ii*) consider these movement data as a timeseries^23^ amenable to further statistical analyses. (According to Wikipedia, upcycling, or creative reuse, is the “process of transforming by-products, waste materials, useless, or unwanted products into new materials or products of better quality or for better environmental value” (https://en.wikipedia.org/wiki/Upcycling)).

We discover that deleterious stochastic signatures in early life manifest in flatter, less rapidly rising developmental trajectories, and they are especially pronounced for High Risk (HR) relative to LR infants. In particular, during sleep, HR infants regardless of age exhibited movement signatures that tended towards the more random, Exponential distribution, with increased noise-to-signal levels relative to LR infants. Strikingly, however, in contrast to LR 1–2 mo-olds who showed significantly more noisy and random movements during a language listening task relative to when they were sleeping, we detected a lack of diversity between “sleep” and “listen” movement signatures in HR 1–2 mo-old infants. Indeed, at 9–10 months, HR infants continued to show the highest noise-to-signal levels in their movements while sleeping, relative to when they were listening to speech, as well as relative to LR infants in both conditions. Antecedents of functionally critical milestones of atypical development (as conventionally conceived) in humans can be quantified as early as 1–2 months after birth.

## Results

**Figure 1:**
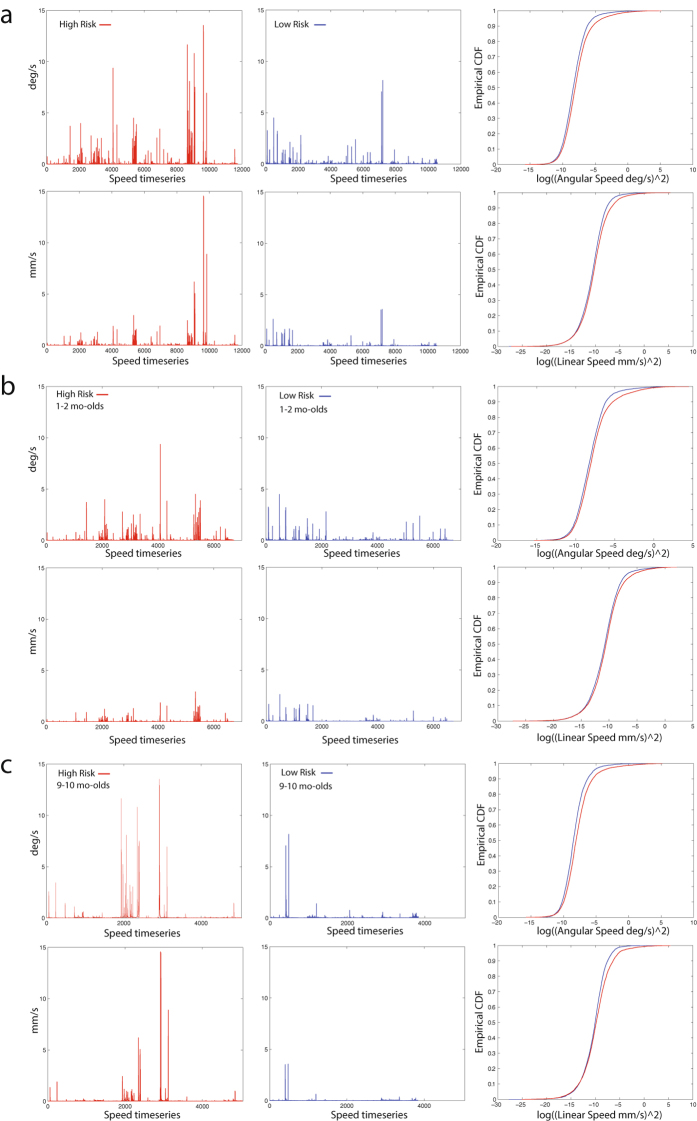
Significantly increased subtle fluctuations in spontaneous head movements as timeseries for High Risk (HR) compared to Low Risk (LR) infants, for angular and linear speed. (**a**) comprises a total of N = 93 datasets (N = 49_HR_ and N = 44_LR_) across all time points, including N = 22 infants tested longitudinally, (**b**) shows data for 1–2 mo-old infants (N = 28_HR_ and N = 28_LR_), and (**c**) shows data for 9–10 mo-old infants (N = 21_HR_ and N = 16_LR_). The right-most panels show empirical Cumulative Distribution Functions (eCDFs). The eCDF of the HR distribution was significantly different from the LR eCDF across the entire sample (angular speed: Kolmogorov-Smirnov, p = 1.7935e-28; linear speed: K-S, p = ﻿6.5195e-18) as well as separate for each age group (for 1–2 mo-olds, angular speed: K-S, p = 1.0415e-10; linear speed: K-S, p = 2.4635e-05 and for 9–10 mo-olds, angular speed: K-S, p = 1.3533e-27; linear speed: K-S, p = 3.7688e-15).

In the first year of life, infants at High familial Risk for developing ASD show significantly increased spontaneous fluctuations of the head relative to LR infants (Fig. 1 illustrates raw angular and linear speed), with the HR distribution significantly different relative to LR (eCDF angular speed: two-sample K-S, D = 0.0761, p = 1.7935e-28; eCDF linear speed: two-sample K-S, D = 0.0601, p = 6.5195e-18; Fig. 1a). This trend held when considering data according to each age subgroup. Specifically, eCDF of HR 1–2 mo-olds was significantly different compared to eCDF of LR 1–2 mo-old infants (N = 28_HR_ and N = 28_LR_) for both angular (two-sample K-S test, D = 0.0592, p = 1.0415e-10) and linear (two-sample K-S test, D = 0.0409, p = 2.4635e-05) speeds (Fig. 1b), and eCDF of HR 9–10 mo-olds was significantly different relative to eCDF of LR 9–10 mo-olds (N = 21_HR_ and N = 16_LR_) for both angular (two-sample K-S test, D = 0.1195, p = 1.3533e-27) and linear (two-sample K-S test, D = 0.0880, p = 3.7688e-15) speeds (Fig. 1c). (For raw data from two individual 1–2 mo-old infants, HR and LR, and their corresponding EPI images, see Supplementary Figure 1). HR infants’ distribution is characterized by significantly higher noise-to-signal levels and increased randomness in subtle fluctuations of the head, relative to LR (non-overlapping 95% CIs, Supplementary Figure 2a).

**Figure 2:**
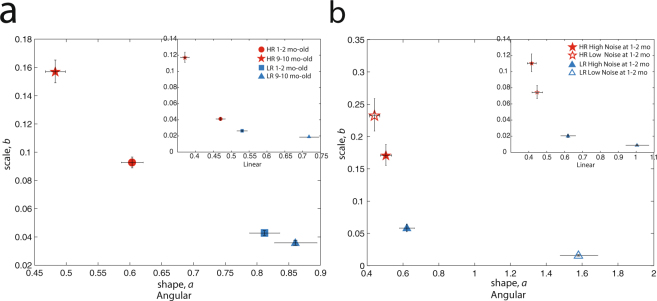
(**a**) HR infants in each age group, 1–2 months (N = 28) and 9–10 months (N = 21), show increased noise-to-signal levels (higher *b* parameter) and increased randomness (toward more Exponential distributions, lower *a* shape parameter) relative to age-matched LR infants (1–2 months: N = 28, 9–10 months: N = 16). (**b**) Considering only 9–10 mo-olds in the longitudinal subset (total N = 22, 11 HR and 11 LR) subgrouped by their noise-to-signal levels as 1–2 mo-olds, LR infants are consistently closer towards more normative ranges on the shape parameter and have lower noise-to-signal levels. Error bars denote 95% CIs.

Examining HR and LR data separately for 1–2 mo-olds and 9–10 mo-olds (cross-sectional: Fig. 2a; Supplementary Figure 2b; consistent trend for longitudinal subset: Supplementary Figure 2c) we find that LR infants’ signatures are more Gaussian and have higher signal-to-noise levels at the end of the 1^st^ year, whereas HR infants’ signatures remain in noisier locations on the Gamma plane (non-overlapping 95% CIs). (This pattern holds for HR infants with a known full-term birth status; Supplementary Figure 3).

### By noise levels at birth

In the longitudinal subset, we confirmed that this overall pattern of more noisy, less symmetric signatures in spontaneous movements holds in HR 9-10 mo-olds regardless of noise-to-signal levels after birth. For both angular and linear speeds, LR infants with the lowest levels of noise after birth had the most normative signatures at the end of the first year relative to LR infants with higher levels of noise, as well as relative to all HR infants (Fig. 2b; non-overlapping 95% CIs).

Examining individual parameter estimates regardless of risk status, we found that infants with higher noise-to-signal levels (*b* parameter, y-axis) also had more random, noisy signatures (*a* parameter, x-axis, tending towards Exponential distributions) in their spontaneous head fluctuations, following a power-law relation f(x) = a*x^b^ (Supplementary Figure 4; Supplementary Table 1 presents goodness-of-fits across and within risk groups, as well as for each age group). Note that on the Gamma parameter plane, lower values on the x-axis (shape *a* parameter) are associated with higher values on the y-axis (scale *b* parameter) (Supplementary Figure 4). We found that HR infants’ *a* vs. *b* fits on angular speed significantly deviated from linearity relative to those of LR infants: Kruskal-Wallis, χ^2^ (1, N = 93) = 68.81, p = 1.08503e-16. A significant between-group difference was also detected in each age group: 1–2 mo-old HR infants’ *a* vs. *b* fits significantly deviated from linearity relative to those of 1–2 mo-old LR infants: Kruskal-Wallis, χ^2^ (1, N = 56) = 41.26, p = 1.33053e-10, and 9–10 mo-old HR infants’ *a* vs. *b* fits significantly deviated from linearity relative to those of 9–10 mo-old LR infants: Kruskal-Wallis, χ^2^ (1, N = 37) = 26.53, p = 2.59972e-07 (consistent for linear speed, all p < 0.001; Supplementary Figure 4a–c).

Overall, these findings establish the presence of atypical signatures of spontaneous movements during rest in HR infants, as early as 1–2 months of age. In addition, we found that the HR infant group shows an atypical Mullen-defined developmental trajectory, with shallower slope and non-overlapping 95% CIs relative to the LR group (Supplementary Figure 5; consistent for Vineland-defined trajectory), prompting us to explore whether atypical behavioral patterns are detectable in independent samples.

### Independent evidence for atypical developmental patte﻿rn in HR﻿ infants

Our external validation analysis of data from 1,445 infants (~N = 945 HR and ~N = 500 LR) from representative UK and US high risk infant-sibling studies^25–28^ confirms an atypical pattern of functioning as ascertained on Mullen and Vineland in HR infants, whether or not HR infants developed ASD later in life— specifically, *lower scores* relative to LR infants (Supplementary Figure 6). In particular, these data show non-overlapping 95% CIs of the mean of Mullen Early Learning Composite (ELC) score at a 2-year follow up as well as non-overlapping 95% CIs of the mean of Vineland Adaptive Behavior Composite (ABC) score at a 7-year follow up for HR ASD-negative siblings relative to LR infants; HR ASD-positive siblings showed the lowest scores. We estimate that up to ~68% of HR infants may present with atypical functioning in childhood, as ascertained using conventional observational instruments.

Given this background, we used a data-driven approach to group infants using their individually defined developmental trajectories. This approach permitted us to study whether trajectories on observational instruments can be predicted via infants’ stochastic signatures at 1–2 (and/or 9–10) months after birth.

### By early learning developmental trajectory

**Figure 3:**
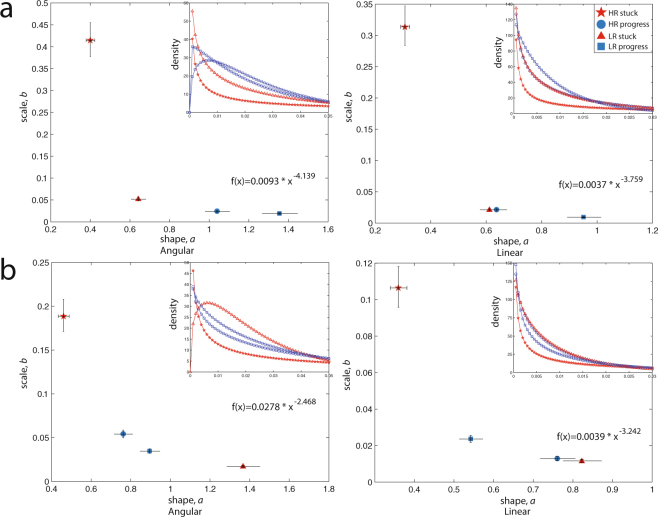
Linking the rapidity of development with stochastic signatures in infants at a biologically High and Low Risk for developing autism later in life. Parameter estimates from movement data during a resting-state sleep fMRI shown for (**a**) 9–10 mo-olds: N = 14_﻿H﻿R﻿_, N = 13_﻿LR﻿_, and (**b**) 1–2 mo-olds: N = 13_﻿HR﻿_, N = 15_﻿LR﻿_. Insets show corresponding Gamma probability density functions (PDFs). High Risk (HR) infants with the most delayed (‘stuck’) trajectory show the most deleterious signatures, with their *shape* parameter tending towards the left on the x-axis (towards more Exponential range), and *scale* parameter emerging higher on the y-axis (towards increased noise-to-signal or fano factor levels), a relation quantified by power law fits (shown) to the data. For all plots, HR infants who do not make rapid developmental progress show more heavy-tailed, less symmetrical PDFs. *Note*. Each individual infant’s *developmental trajectory is defined independently in a data-driven manner*, using exponents of the individual power fits to score data comprising at least 2 or more Early Learning Composite (ELC) scores (for infants with available Mullen scores) collected longitudinally (at 6, 12, and/or 18 months) on the Mullen Early Learning scale. For each risk group, infants are subgrouped into a progress group (those with a higher, more rapidly rising exponent/slope) and a flatter or ‘stuck’ group (those with a lower exponent, indicating a slower rise over time). For Mullen ELC and 9–10 mo-olds data, HRstuck: N = 7_ELC_, HRprogress: N = 7_ELC_, LRstuck: N = 7_ELC_, LRprogress: N = 6_ELC_. For Mullen ELC and 1–2 mo-olds data, HRstuck: N = 6_ELC_, HRprogress: N = 7_ELC_, LRstuck: N = 7_ELC_, LRprogress: N = 8_ELC_. Error bars denote 95% CIs.

Figure 3 shows stochastic signatures of infants subgrouped by the rapidity of their developmental trajectory on the Mullen ELC score (Fig. 3a shows 9–10 mo-olds and Fig. 3b shows 1–2 mo-olds). (Physiological data shown are from infants who had at least 2 time points on the Mullen at 6, 12, and/or 18 months). We found that 9–10 mo-old HR infants who were ‘stuck’ on their ELC trajectories showed the highest noise-to-signal levels (*b* parameter) and the most random shape of distribution (towards Exponential, *a* parameter) in their head movement fluctuations relative to all other subgroups (non-overlapping 95% CIs, consistent for both angular and linear speeds), a pattern quantified by power-law relations (all R^2^ > 0.99). (Supplementary Table 2 presents goodness-of-fit results for power as well as exponential fits). These laws ascribe increased rapidity with which general development occurs with more normative spontaneous movement signatures.

Examining the relation between proximal or near future developmental trajectory and movement signatures in 1–2 mo-olds (Fig. 3b), we again found that HR infants who had gone on to have the most delayed (‘stuck’) developmental trajectory showed significantly worse signatures relative to all other infants on both angular and linear speeds (non-overlapping 95% CIs; Supplementary Table 3 presents goodness-of-fit results). (For both age groups, a consistent pattern is obtained when using normalized peaks data; Supplementary Figure 7). (In exploratory analyses using available Vineland ABC scores, we confirmed this pattern, detecting that 9–10 mo-old HR infants with the lowest ABC scores have the most deleterious movement signatures; Supplementary Figure 8).

### By condition: dissociation of movement signatures during “listening to language” and “sleep” tasks

We next asked whether movements in general are noisier in HR infants or whether stochastic signatures during sleep are dissociable from those during behaviorally relevant periods of time (i.e., when the infant is likely learning the statistics associated with sensory stimuli), and looked for evidence of an age-related pattern. We examined movement signatures of 1–2 mo-olds and 9–10 mo-olds as they were listening to native language speech during a functional MRI scan, as compared to simply resting or sleeping during the resting-state fMRI scan.

**Figure 4:**
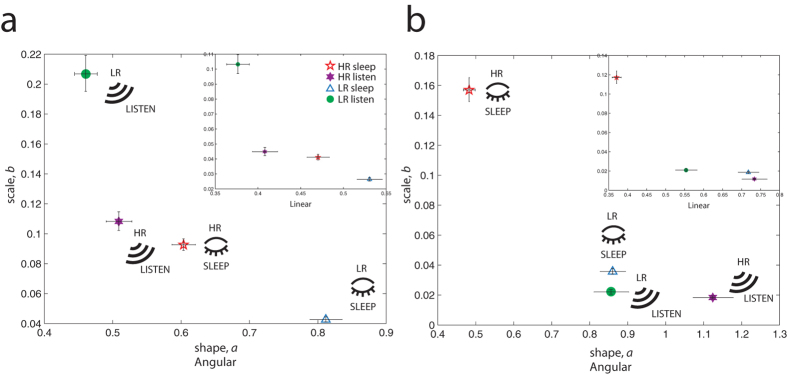
Dissociation of movement signatures to “listen” vs. “sleep” conditions as a function of risk status and age. (**a**) At 1–2 months, LR infants during sleep show the most symmetric signatures with high signal-to-noise levels yet the noisiest, least symmetric signatures during wakefulness when listening to native language. HR infants show more similar movement signatures during the two conditions. (**b**) At 9–10 months, HR infants show heightened noise levels during sleep and lower levels during the listening task relative to LR infants. *Note*. Data points during sleep are the same as those presented in Fig. 2a and included here for ease of comparison relative to those from the listening task. Native Language listening, 1–2 mo-olds: N = 27_HR_, N = 28_LR_; 9–10 mo-olds: N = 19_HR_, N = 14_LR_. Sleep, 1–2 mo-olds: N = 28_HR_, N = 28_LR_; 9–10 mo-olds: N = 21_HR_, N = 16_LR_. Most of the infants who were scanned during sleep at either time point were also scanned in a separate scanning session while listening to native language speech. Error bars denote 95% CIs.

Strikingly, we found that 1–2 mo-old infants at High familial Risk for developing ASD do not show the same diversity in their head movements during distinct behavioral conditions as do LR infants (cross-sectional: Fig. 4a; consistent trend for longitudinal subset: Supplementary Figure 9a). Note that 1–2 mo-old LR infants showed the most normative motor patterns during the sleep scan (data also shown in Fig. 2a), but significantly noisier movement patterns while listening to language (Fig. 4a) (non-overlapping 95% CIs). In contrast, 1–2 mo-old HR infants’ movement signatures during sleep and during listening to language conditions were more similar, with nearly overlapping scale parameter. At 1–2 months, HR infants moved in a similar way whether or not they were sleeping or exposed to acoustic stimuli—human language.

This pattern held when examining individual infant fits, with significant differences between sleep and listen conditions in LR infants on both shape and scale parameters: *a* shape (Wilcoxon signed rank test: p = 0.0021; LR sleep median: 1.9557; LR native language median: 0.7418) and *b* scale (Wilcoxon signed rank test: p = 0.0059; LR sleep median: 0.0097; LR native language median: 0.109) but indistinguishable in HR infants on either parameter, *a* shape (Wilcoxon signed rank test: p = 0.442; HR sleep median: 1.1182; HR native language median: 1.3566) and *b* scale (Wilcoxon signed rank test: p = 0.8854; HR sleep median: 0.0312; HR native language median: 0.0247).

A different pattern emerged in 9–10 mo-olds: LR infants had more similar signatures during the two conditions, while HR infants had abnormally lower (angular speed, non-overlapping 95% CIs) noise-to-signal levels when listening to speech. To emphasize, at 9–10 months, HR infants continued to have the worst movement patterns during sleep (same data as Fig. 2a, consistent with our previous findings above) and abnormally low noise-to-signal levels when listening to native language (Fig. 4b; consistent trend for longitudinal subset: Supplementary Figure 9b).

Could differences between movement signatures to distinct tasks at 1–2 months have measureable effects on movement signatures to those tasks at 9–10 months? As a context-driven test of the perception-action hypothesis, we subgrouped infants in the longitudinal subset who underwent both scans at both time points, grouping individuals by the difference in their scale, *b* (i.e., fano factor, FF) parameter on the resting-state and language scans (we obtained similar subgroupings when using shape, *a* parameter), and then examining their signatures at either resting or listening to language. 9–10 mo-old infants were subgrouped into a “highDiff” group if their difference as 1–2 mo-olds between native FF and resting FF was positive (“native” FF minus “rest” FF), and “lowDiff” if FF was negative. We found that during a language listening scan, 9–10 mo-old LR infants who had the lowest FF difference as 1–2 mo-olds (lowDiff) had less symmetric and noisier signatures relative to those who had a positive FF (highDiff). In contrast, HR infants who showed the least diversity in their movements as 1–2 mo-olds (lowDiff group) showed abnormally low noise-to-signal levels ﻿and increased symmetry in their movements during the language task (Supplementary Figure 10a). Further, and consistent with the general trend reported above for resting-state scan signatures, 9–10 mo-old HR infants had movements that were characterized by increased noise-to-signal levels and increased randomness during the sleep resting-state scan, regardless of differences in noise levels between native and sleep scans as 1–2 mo-olds (Supplementary Figure 10b).

### Biological origins of atypical movement signatures: the role of increased Father’s age

In the final analysis we probed whether those infants conceived from an older father have more deleterious movement patterns relative to infants whose father was young at conception, seeking a mechanistic insight into the nature of the effects reported in the previous sections. Individuals with ASD have been shown to have more deleterious *de novo* (DN) mutations that are functional targets of genes that affect synapse formation (FMRP-fragile X) and chromatin modifiers. In addition, increased numbers of DN mutations in ASD individuals born to older, relative than younger, fathers have been reported^29^ and age-related alterations in sperm DNA methylation have been reported in mice offspring sired by animals of advanced paternal age^30^. It is therefore possible that transmitted DN events or atypical DNA methylation patterns in children of older fathers could adversely affect genes (or expression of genes) that are critical for development of the nervous system. (In our sample of individuals with available data, because mother’s age was significantly correlated with father’s age (rho = 0.6784, p = 2.6839e-04), we focus on the role of father’s age. Future work using larger samples should stratify datasets by mother’s age).

**Figure 5:**
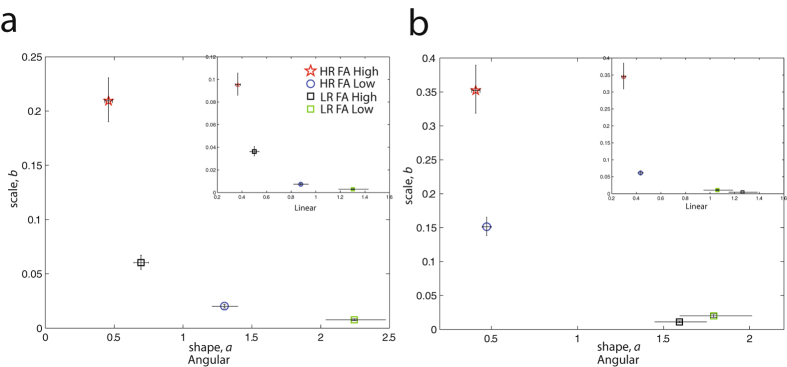
Biological origins of atypical spontaneous movement patterns during sleep: the role of father’s age. (**a**) shows 1–2 mo olds and (**b**) shows 9–10 mo-old infants subgrouped by higher vs. lower Father’s Age (FA) at conception. At both time points, HR infants with an older father (“HR FA High”) have movement signatures characterized by heightened noise-to-signal levels that tend towards Exponential, less symmetric ranges, in contrast to LR infants with a younger father who show the most normative movement signatures. *Note*. For 1–2 mo-olds with available father’s age data, HR_FA High_: N = 6, HR_FA Low_: N = 5, LR_FA High_: N = 4, LR_FA Low_: N = 3, and for 9–10 mo-olds, HR_FA High_: N = 6, HR_FA Low_: N = 7, LR_FA High_: N = 3, LR_FA Low_: N = 2. Error bars denote 95% CIs.

Examining infant movements subgrouped by their father’s age at conception (for a subset of infants with available data, separately for 1–2 and 9–10 mo-olds), we found that across both time points, signatures were least symmetric and with higher noise-to-signal levels for HR infants with older (“Father’s Age High”), relative to younger fathers (“Father’s Age Low”), and most symmetric with higher signal-to-noise levels for LR infants with the youngest fathers (Fig. 5). Specifically, HR_FAHigh_ 1–2 mo-old infants have more deleterious signatures relative to HR_FALow_ infants (non-overlapping 95% CIs for noise-to-signal scale *b* and shape *a* parameters for both angular and linear speeds). A similar pattern was observed for LR infants: LR_FAHigh_ 1–2 mo-old infants show worse signatures relative to LR_FALow_ infants (non-overlapping 95% CIs for noise-to-signal scale *b* and shape *a* parameters for both angular and linear speeds). We also note that LR_FAHigh_ have more deleterious signatures relative to HR_FALow_ infants. Considering movements of 9–10 mo-olds, HR_FAHigh_ infants showed the most deleterious noise-to-signal (*b* parameter) levels relative to HR_FALow_ infants (non-overlapping 95% CIs for both angular and linear speeds) and both LR subgroups that showed more symmetric signatures with increased signal-to-noise levels. Thus, considering both time points (1–2 and 9–10 months), HR infants conceived with an older father had spontaneous movement signatures during sleep that were characterized by the highest noise levels with the most randomness.

For infants with available native language and father’s age data (1–2 mo-olds only) we detected an advanced father’s age-related pattern that is consistent with our earlier sensorimotor patterns accompanying speech perception. 1–2 mo-old LR infants with the youngest father (LR_FALow_) showed the least symmetric patterns with higher noise-to-signal levels relative to LR_FAHigh_ infants (non-overlapping 95% CIs for noise-to-signal *b* and shape *a* parameters for both angular and linear speeds); HR infants showed more symmetric and the least noisy signatures regardless of their father’s age (Supplementary Figure 11a). Note that sensorimotor patterns of 1–2 mo-old LR infants with the youngest father were the most context-flexible of all subgroups; that is, LR_FALow_ had the most symmetric and least noisy patterns during sleep and the least symmetric and most noisy during wakefulness (Supplementary Figure 11b).

## Discussion

We report the first evidence that infants at High familial Risk for developing ASD have atypical head movement signatures relative to LR infants during 1^st^ year of life. We found significant quantitative differences between HR and LR infants’ movement signatures as early as 1–2 months after birth, observed across 2 different contexts—during rest, when the infants were sleeping, and when they were listening to native language.

Our key finding is that the noisiest and most random signatures of spontaneous movements in HR infants are present during rest (resting-state sleep fMRI scan), a state during which infants spend the majority of their time during the 1^st^ year of life (Fig. 2a) (results consistent for 1–2 and 9–10 mo-olds in the longitudinal subset, Supplementary Figure 2c). In the longitudinal subset, we found that 1–2 mo-old LR ‘low-noise’ infants—those with the most normative signatures at birth during a resting-state scan—showed the most normative signatures at 9–10 months, whereas HR 9–10 mo-olds continue have deleterious patterns compared to peers regardless of noise levels at 1–2 months (non-overlapping 95% CIs), a pattern consistent for both angular and linear speeds (Fig. 2b). Importantly, these atypical patterns are functionally linked to flatter development trajectories measured on the Mullen Early Learning Composite score, a trend quantified by a power law relation (Fig. 3). In the earliest age group studied, we find that HR 1–2 mo-olds who went on to have the least normative trajectory of early development—those who are ‘stuck’—show the most deleterious stochastic signatures in their spontaneous head movements (Fig. 3b).

We detected a dissociation between movement signatures in HR and LR infants during sleep vs. native language conditions that interacted with age. Surprisingly, at 1–2 months, LR infants showed increased noise-to-signal levels and more randomness in their head movements relative to HR infants while listening to language compared to sleeping, but this was not the case for 1–2 mo-old HR infants, whose movements were more similar between the two conditions (Fig. 4a). Our findings speak to work by Dehaene-Lambertz and colleagues^31^ who found greater pre-frontal activation in 2- to 3- mo-old healthy infants while listening to forward vs. backward recordings of native speech during awake periods, relative to when infants were sleeping. In our study, the presence of distinct sensorimotor patterns of 1–2 mo-old LR infants during speech (vs. sleep) suggests that LR infants are alert and reactive as the developing brain learns to process human speech and as speech production commences. These data are consistent with the role of innate, evolutionarily-driven and higher-level knowledge that helps constrain ‘lower-level’ perception (cf. in refs 32 and 33). In this sense, 1–2 mo-old LR infants’ signatures reveal attunement to evolutionarily socially important auditory input, supporting the idea that (normative) development does not proceed in a bottom-up manner.

By 9–10 months, HR infants showed the highest noise-to-signal levels and most random movement signatures during sleep, while also showing abnormally low levels of noise and randomness in movements when listening to language (Fig. 4b). This developmental change in movement signatures of 9–10 mo-olds to “sleep” vs. “listen” conditions is confirmed in the longitudinal subset (Supplementary Figure 9b). Further, we found that 9–10 mo-old HR infants with the smallest differences between their native and resting FFs as 1–2 mo-olds had abnormally low levels of noise (i.e., their signatures had high signal-to-noise levels and most symmetric features) while awake and listening to human language (Supplementary Figure 10a), with an opposite pattern for 9–10 LR infants whose signatures were similar as 1–2 mo-olds, showing low signal-to-noise levels and least symmetry. As noted in Kuhl *et al*., 2014, utterances of typically developing infants begin to resemble “the fully resonant nuclei that characterize speech” around 3 months, while at 5 months, infants “imitate pitch patterns”^14^. Further, a typically developing motor system is functionally important during early language acquisition and contributes towards an appropriate “commitment” to native language by 11–12 months^14^.

Our probe into the putative biological origins of these atypical movement signatures revealed a role of increased father’s age at the time of conception. HR infants conceived with an older father (~7 years older on average) have the highest noise-to-signal and significantly random movements during sleep of all other infants (Fig. 5). Considering sensorimotor patterns during the native language scan and resting scan as a function of father’s age (Supplementary Figure 11b), 1–2 mo-old LR infants with the youngest fathers had the most context-responsive patterns of all subgroups: the most symmetric and least noisy patterns during sleep, and the least symmetric and most noisy patterns while listening to language. We consider these findings as follows.

Because of continued sperm cell division during a male’s lifetime, older fathers may contribute lower quality germ line. Increased paternal age is associated with an increase in the number of *de novo* (DN) mutations transmitted to the offspring (“1–3 fold increase in the number of *de novo* SNVs for ten years of paternal age”^29^) as well as an aberrant sperm DNA methylation which may exert prenatal influence on which genes are expressed in the developing fetus. DNA methylation is a process that governs how genes are “switched” on or off, thereby controlling gene expression during early development. Normally, male sperm DNA methylation is “reprogrammed” after fertilization^30^, but recent work using advanced paternal age mice models has provided evidence that older fathers contribute an abnormal methylation pattern characterized by reduced sperm DNA methylation at CpG islands^30^. Either paternal accumulation of DN mutations or paternal transmission of DNA methylation abnormalities could affect genes or expression of genes that are important during development of the nervous system and key brain structures, including those supporting cerebellar function^30^. In turn, atypical functioning of these genes (for example, those that govern synapse formation) could in principle manifest in movement signatures in HR infants with older fathers, which we observe in the current work. Inappropriate synapse formation and function during a time of rapid brain development^34^ may facilitate production of further atypical movement signatures as early as 1–2 months in human neonates, which we detect here. As the origins of the atypical movement signatures may be prenatal and at least partially due to relaxed selection in the population, our results highlight the sensibility to cold-store male sperm (e.g., ref. 35) for later use.

Thus, in HR infants, we find atypical sensorimotor patterns in response to distinct information streams during a critical postnatal period, with important developmental (including linguistic) milestones to be achieved during the intervening period between 1–2 and 9–10 months—the two time points at which we derived movement data. A very early difference in sensorimotor experience may arguably make it more difficult for infants to appropriately and optimally perceive (i.e., capture) acoustic and visual input during social exchanges with parents and caregivers and may yield less precise, uncertain estimates of desired or ‘legal’ speech patterns, making it more difficult to create internal models that serve as effective representations of the physical world.

Another account for the result of the present study is that there were differences not in the sensorimotor patterns *per se* but in the transition of behavioral (or sleep) states in 1–2 mo-old HR infants. For example, given that the previous studies suggested that sleep disorder is related to ASD^36^, movement signatures that were obtained in this study may be dependent on the states (e.g., ref. 37) even within a single infant. While in this work, ‘sleep’ state is defined as an infant having their eyes closed, in future work it would be important to code sleep states during rest (sleep) in HR infants, perhaps over longer periods of time, in order to investigate the role of physiological states in the emergence of atypical development in humans.

As a final possibility, at a different level of analysis, HR infants may have an aberration in the functioning of the innate, top-down processes that normally assist in constraining lower-level processes in infancy (cf. ref. 32). Unlike LR infants, 1–2 mo-old HR infants seem to be less attuned to native language auditory inputs, as inferred from their movement signatures. In this sense, 1–2 mo-old HR infants may be said to operate within an “altered neural space”, and thus generate hypotheses given that altered space^32^. The finding of less diverse signatures during the two conditions suggests that HR infants function within a restricted hypothesis space very early in life. Such early, possibly innate, differences in infants’ sensitivity to ambient information will likely have downstream consequences for cognitive organization. While for individual infants the degree of difference may vary, the resulting variability would be consistent with research documenting extreme phenotypic heterogeneity in toddlers (and older children) who receive ASD diagnoses or those presenting with atypical (non-ASD) development.

Given our current findings, we propose a Developmental Optimality Constraint Helix concept of early development in humans that conceives social, emotional, and linguistic difficulties (the hallmark core symptoms of ASD conventionally diagnosed no earlier than 2 years) to be downstream consequences (or epiphenomena) of suboptimal capacity to appropriately parse sensory input at or near the time of birth. This concept holds that physiological features (such as those affecting motor system fidelity) and cognition are inextricably linked in early life: both are bootstrapped by the evolution (cf. ref. 38) toward developing constraints (abstract knowledge) when building appropriate (e.g., capable of tolerating normative amounts of uncertainty) internal models for effective interaction with the environment.

Here we show that extracting important structure in the underdetermined information stream may be impeded, for some infants, by the presence of context-inflexible movement signatures (or inflexible signatures when transitioning between behavioral states), as early as 1–2 months after birth. From this perspective, the topology of early development in humans has important implications for objective understanding of extremely heterogeneous neurodevelopmental disorders, including ASD. The current study thus begins an important effort to understand how this occurs in early life and outlines several (not mutually inconsistent) accounts of the present findings that require additional investigation.

Importantly, we falsify the theory that infants at High familial Risk for developing ASD are generally expected to develop normally. We show this with Mullen and/or Vineland profiles as a function of age in the current dataset (Supplementary Figure 5) and data from 1,445 infants in 4 independent datasets (Supplementary Figure 6), as well as with movement signatures during rest that do not improve with age (Fig. 2). We establish that quantitative signatures of atypical development in HR infants can be detected as early as 1–2 months of age (e.g., Figs 3b and 4a). Improved understanding of (varied) neurobiological mechanisms underlying atypical development in humans can thus be informed by quantitative subtyping during the 1^st^ year of life, which may in turn produce neurobiologically informed biomarkers and lead to targeted therapeutic approaches for affected individuals.

### Implications for infant brain imaging research

Our data refute the implicit assumption that no differences exist between movement signatures as a function of autism risk status in infants as early as 1–2 months of age. On the contrary: our work establishes that HR infants move significantly differently relative to LR infants while being scanned. Our report of significant between-group differences invites a rethinking for the field of infant brain imaging. This point is relevant, as the original purpose of measuring BOLD signal in these infants may be to examine brain activation and/or functional connectivity. Indeed, if such information were available and valid, comparisons with head movements might have provided a richer picture to understand the sensorimotor patterns of infants. However, BOLD obtained from functional MRI^39^ (as well as T1- and/or T2-weighted images obtained from structural MRI (e.g., ref. 40) as well as other modalities including diffusion-weighted and/or tensor imaging) is susceptible to movement-induced artifacts that mimic high-level effects of interest. For example, greater movement at the between-group level, even after “scrubbing”, may produce a finding of reduction of longer-range connectivity in the group that moved more.

Such effects cannot be easily corrected either using prospective (i.e., during acquisition) or retrospective motion correction (e.g., ref. 39) (but see ref. 23 approach for ASD that involves reduction of sample size and objective group matching including non-linear head movements in fMRI). In the current work, excluding a large proportion of infants with excessive movement using an objective approach such as in ref. 23 would substantially reduce sample size; cleaning and hand-selecting segments of usable data is a subjective approach that may raise doubts about the validity of subsequent statistical brain-based inference with such data.

As an example of these challenges in the context of structural MRI, consider recent work by Lewis and colleagues^41^ who scanned HR and LR infants during natural sleep and applied graph theory metrics to diffusion-weighted images, either in common stereotaxic or native space. The authors reported that HR ASD-positive infants have reduced network efficiencies with regard to, in particular, lower-level sensory regions (relative to the “non-ASD” group which comprised both HR ASD-negative and LR ASD-negative infants). Yet, a pattern of between-group difference in the reduction direction is exactly what would be predicted if the HR ASD-positive group moves more relative to the comparison group.

In particular, the authors noted that if the quality checks for diffusion images “excluded > 20% of the directions for any size subset of the largest *b*-values”, then the entire dataset was excluded from the analyses. However, it could be the case that datasets of ASD-positive infants have a consistently smaller number of included directions relative to those of non-ASD infants (e.g., LR ASD-negative infants); that is, without reaching the threshold for individual datasets, the entire ASD-positive sample nonetheless could be of poorer quality and affect indices of connection length. An additional concern is that the authors estimated connection length using diffusion-weighted images normalized to participants’ native space; as participants’ T2 structural images were acquired sequentially relative to the diffusion images, unique or common motion patterns accrued in this different modality could compound the influence of movement on MRI data from a given participant. Hence, between-group differences in movement (and/or male to female ratio: see Table 1 in ref. 41), and not underlying brain structure, could drive ASD vs. non-ASD differences on the metric that indexes “connectivity”.

On a related but a more provocative note, our data speak to previous findings in HR infant-sibling studies whose data document extreme variability of measure outcomes in HR infants relative to LR infants. For example, Jones and Klin^27^ present a positive view of their data from an eye-tracking study, presenting an interpretation that “attention to eyes is present but in decline” in 2 mo-olds who later develop ASD. However, a close examination of their data reveals atypically elevated intercepts of the % of fixation time to the eyes for ASD-positive infants, a pattern that also held for HR infants who presented with a “broader autism phenotype” and HR ASD-negative infants (see Extended Data Figure 6 in ref. 27). Thus, these HR infant data are atypical (also note wider 95% CIs intervals around the slopes, suggesting increased variability of the fits) regardless of whether the infants went on to present with a clinical diagnosis of ASD as toddlers. That is, HR infants’ eye-looking baseline as 2 mo-olds is already not at the same level as that of LR infants.

Another example is provided in the study by Hazlett and colleagues^28^, whose findings of increased total brain volume (TBV) in HR ASD-positive infants between 12 and 24 months relative to HR ASD-negative infants and LR infants are explained by the higher prevalence of males receiving ASD diagnoses in the HR ASD-positive subgroup. When male-only data were examined (see Extended Data Table 8 in ref. 28; N = 13 in the ASD-positive group), between-group differences were no longer significant. Of relevance to our discussion, note that the standard error (SE) of the TBV slopes is not only higher in HR infants relative to LR infants in general but it is higher within the subgroupings of HR infants. Specifically, the SE of the TBV is 6.79 times higher for HR ASD-positive vs. HR ASD-negative infants when using 2^nd^ year trajectory values as well as 4.67 times higher when using trajectory values at 24 months. Taken together, these observations highlight that sources of individual variability (i.e., sex, movement signatures, risk status) may substantively affect between-group level inference, including brain connectivity measures, in ASD infant-sibling studies.

### Open Science approach for infancy research

The measurement of movement signatures to assess developmental disorders can be performed in a much less expensive manner and at higher temporal resolution, using motion sensors and/or video analysis^42^, but the current approach used in this study attempts to highlight a source of complementary and new insights in conducting infancy research in the spirit of Open Science. Over the past several decades, MRI scanner manufacturers and researchers have formulated standardized protocols for neuroimaging data acquisition, including minimizing excessive movement of the head during the scan (e.g., by cushioning participants’ heads). In addition to restricting head movement, the functional MRI data are acquired under well-controlled conditions using fixed temporal resolution (usually every 2 or 1.5 seconds per acquired volume). Further, modern MRI facilities include an in-bore video feed of the participant that can be used to monitor eye status (open or closed eyes) and some acquire respiration and/or heart rate data in tandem during the scan. Thus, these data are collected under very similar conditions worldwide, providing a unique and unprecedented opportunity for researchers across disciplines. In addition to relying on existing open-access databases and datasets from publications (e.g., http://www.datadryad.org), researchers can collaborate with neuroimaging colleagues at their institutions to obtain movement parameters for a specific sub-population of interest, including infants with congenital heart problems and premature infants, as well as fetuses—any of the domains in fetal and infant research that acquire functional MRI scans of their participants.

## Conclusion

Infants at High familial Risk for autism are at risk for atypical development later in life. HR infants who showed the most flat or less rapidly rising developmental trajectory as toddlers had increased noise levels and decreased symmetry features in their resting-state sleep fMRI movement signatures as 1–2 mo-olds. We discovered striking context-inflexible signatures in 1–2 mo-old HR infants, who showed similar signatures during a language listening task and during rest. Our findings falsify the theory that HR infants develop in a typical manner and provide the earliest evidence to date that signatures of atypical development in HR infants are quantifiable as early as 1–2 months after birth. These findings may be alternatively accounted for by early sensorimotor differences, differences in transitioning between behavioral or sleep states, and/or altered neural hypothesis space that affects development in HR infants. Our current work raises new, provocative and exciting questions for future infancy research, and provides immediate clinical guidance for pediatricians to watch all HR infant siblings for signs of atypical development.

## Methods

### Experimental Design

Data used in the preparation of this study were obtained from the NIH-supported National Database for Autism Research (NDAR). NDAR is a collaborative informatics system created by the National Institutes of Health to provide a national resource to support and accelerate research in autism. The dataset identifier (along with the Submitter) is NDARCOL0002026 (Susan Bookheimer). Our inclusion criteria for selecting specific NDAR study-sites consisted of (*i*) availability of at least 1 resting-state or functional scan; data in original format with no motion correction or other post-scan processing applied, (*ii*) ages from birth to around 1 year during the scan (*iii*) availability of additional neuropsychological and clinical assessments between 0 and approximately 2 years, (*iv*) inclusion of participants at high risk for ASD, (*v*) at least 10 participants satisfying *i-iv*. These criteria yielded the ACE UCLA study-site population, which is the focus of the current work. All available rs-fMRI and fMRI native language datasets from all infants were analyzed. These data comprise datasets included in the NDAR annual data release as of May 2017.

Data are de-identified in compliance with the U.S. Health Insurance Portability and Accountability Act (HIPAA) guidelines. Signed written informed parental consent was obtained by original study investigators in accordance with U.S. 45 CFR 46 and the Declaration of Helsinki for participation; IRB-approved research protocols included neuroimaging and clinical assessments. Analyses of these de-identified data were reviewed and approved by, and a waiver of informed consent was obtained from, the Institutional Review Board of Columbia University Medical Center.

Healthy, full-term infants who met inclusion criteria (normal pregnancy with no complications and birthweight > 3,000 g) during recruitment at the Autism Center of Excellence at UCLA were included in our sample of participants. Post-hoc examination of full-term birth status for infants for whom this information was available (N = 36) revealed a small number of infants (N = 3 from the HR group and N = 1 from the LR group) whose gestational age was less than 37 weeks; we repeated key analyses with full-term HR and LR infants.

The sample included 71 unique infant participants with MRI scans and behavioral and clinical assessments. All 71 participants underwent a resting-state scan (rs-fMRI) during natural sleep conditions (note that as the scans were not collected for clinical purposes, no sedation during scanning was used during any of these scans, for any of the participants). Here, ‘sleep’ is defined as an infant lying in a quiet state, having their eyes closed; it is possible that some infants could be in a state of quiet wakefulness for intermittent periods during the scan (note that even with eyes closed, ascertaining sleep state (i.e., active or quiet) is challenging during an MRI scan and should be monitored in future studies using eyelid status and/or respiration and heart rate).

In addition, 69 of these infants also underwent a functional MRI (fMRI) scan during which infants listened to recordings of human speech in their native language (note that as the language scan was shorter and fewer datasets were available across diverse age groups (for 1–2 and 9–10 mo-olds), in the current work we present data from both but primarily focus on movement data derived from rs-fMRI scans).

A total of 181 datasets (93 for resting-state and 88 for native language scans) were available for these 71 unique participants across age groups of 1–2 mo-olds and 9–10 mo-olds. (Out of the 71 unique participants, N = 49 were scanned at only one time, at approximately 1–2 months or 9–10 months, whereas N = 22 comprised the longitudinal imaging subset and underwent two resting-state scans at these two time points, for a total of N = 93 resting-state fMRI datasets). From the overall sample, N = 38 were infants at “High Risk” (HR) for developing Autism Spectrum Disorders (ASD); High Risk is defined exclusively as a participant who has a biological sibling diagnosed with ASD (N = 33 were “Low Risk”, LR). At 1–2 months of age, a total of N = 28_HR_ and N = 28_LR_ infants were scanned during a resting-state scan and at 9–10 months, a total of N = 21_HR_ and N = 16_LR_ infants were scanned.

Overall for this dataset (N = 71), the Male to Female ratio (M/F) is 43 M/28 F (ratio by risk status: HR (23 M/15 F) and LR (20 M/13 F)). Participants’ ages at the time of their first scan are as follows: N = 25 (1mo), N = 31 (2mo), N = 12 (9mo), N = 2 (10mo), N = 1 (11mo); we note that NDAR provides ages in months.

In the longitudinal imaging data subset (N = 22), exactly N = 11 infants were High Risk (6 M/7 F) and N = 11 Low Risk (6 M/7 F) (age at first scan (also included in the above count) was 1 month (N = 10), and 2 months (N = 12); age at second scan: 9 months (N = 20) and 10 months (N = 2)).

Native language scans at 1–2 and 9–10 months were completed by N = 69 of these 71 participants, with N = 19 comprising the longitudinal subset (HR = 10 and LR = 9). Overall, data at 1–2 months were available for N = 55 infants (N = 27_HR_ and N = 28_LR_) and at 9–10 months for N = 33 infants (N = 19_HR_ and N = 14_LR_).

#### MRI acquisition parameters

Resting-state MRI (rs-fMRI) and functional MRI (fMRI) Blood Oxygenation-Level-Dependent (BOLD) data were acquired at a 3 Tesla MR scanner. The rs-fMRI BOLD signal was obtained with a T2*-weighted echo planar imaging (EPI) sequence (repetition time (TR) = 2000 ms (½ Hz temporal resolution), for a total scan time of 8 minutes (240 volumes)). (Specifically, EPI parameters for the rs-fMRI scans were: [TR/TE: 2000/28 ms, flip angle = 90°, FOV = 192 mm, 56 × 56 matrix, 34 axial 4 mm slices]). The fMRI EPI parameters for native language scans were: [TR/TE: 3000/28 ms (1/3 Hz temporal resolution), flip angle = 90°, FOV = 192 mm, 56 × 56 matrix, 34 axial 4 mm slices], with a scan duration of 7.2 minutes (144 volumes).

### Analytic Strategy

#### Extraction of head movements from neuroimaging volume images

Head movements were estimated by pre-processing resting-state MRI image volumes using Statistical Parametric Mapping (SPM8), an open-access, widely used software for processing neuroimaging data (http://www.fil.ion.ucl.ac.uk/spm/software/spm8/) running MATLAB version 8.3 (R2014a) (The MathWorks, Inc., Natick, MA). As head movements affect BOLD signal intensity of collected volumes over time and represent a known confound in neuroimaging^43^ software for processing MRI data commonly include a motion estimation component (in SPM, the ‘realign’ component includes ‘estimate’ and ‘reslice’; the ‘reslice’ function resamples the volumes using estimated motion parameters). In SPM, realignment of scanned volumes involves estimating the six parameters of an affine ‘rigid-body’ transformation (b-splines interpolation using least-squares approach) that minimizes the differences between each successive scan and a reference scan^43, 44^. The default reference scan in SPM8 is the first scan (volume), to which all subsequent volumes are realigned. Of note, the linear (affine) position transformations specified for some portion of the image hold for all other portions of the image^44^ by virtue of the treatment of this problem as one involving rigid body transformations. For each participant, we pre-processed 3D volume image files in the NIFTI format (.nii), separately for resting-state and functional native language scans, producing an output with six motion parameters (3 linear translations in x, y, z directions, and 3 rotations: pitch (about x-axis), roll (about y-axis), and yaw (about z-axis)) which is recorded as an rp_%s.txt file in SPM8. The first three columns of the output file are linear parameters in mm, and the last three columns are rotational parameters given in radians that we converted to degrees (*180/pi).

#### Overview

Natural biophysical processes fluctuate in amplitude and frequency both on the short- and longer time scales. When a signal is measured continuously the timeseries exhibit peaks and valleys, underlying a process whose statistical properties are amenable to empirical interrogation. Speed—a measure of how fast an object is moving per unit of time—during spontaneous, natural movements in humans is one example of such a fluctuating signal. In the current work we convert volume-to-volume head movements to speed and study the statistical features of the signal as a Gamma process, without *a priori* assumptions about the signal’s underlying distribution (i.e., Gaussian or non-Gaussian) using a maximum likelihood estimation (MLE) procedure in MATLAB.

Both linear and angular volume-to-volume speeds were computed as follows. Speed is defined as the linear (or angular) difference (delta) between sequential volumes (in three directions x, y, and z) per unit of time, equal to the inter-scan interval (TR):

1 $$Spee{d}_{volume \mbox{-} to \mbox{-} volume}(Linear)=\sqrt{{({\rm{\Delta }}x)}^{2}+{({\rm{\Delta }}y)}^{2}+{({\rm{\Delta }}z)}^{2}}/TR$$

(e.g., in the case of the x-direction, Δ(delta) designates change between volume ‘2’ and preceding volume ‘1’: ${\rm{\Delta }}x={x}_{i(2)-}{x}_{i(1)}$). Similarly for angular rotations, speed is given by:

2 $$Spee{d}_{volume \mbox{-} to \mbox{-} volume}(Angular)=\sqrt{{({\rm{\Delta }}\alpha )}^{2}+{({\rm{\Delta }}\beta )}^{2}+{({\rm{\Delta }}\gamma )}^{2}}/TR.$$

Linear speed is expressed as mm/second. Angular coordinates were Euler angles and angular speed is expressed in deg/second (as angles were small they were not represented as quaternions). For each participant, we computed linear (mm/second) and angular (deg/second) speed timeseries for each scan session. Timeseries were filtered using a triangular filter with width parameter = 2 in order to study the statistical character of the timeseries as a continuous Gamma process. Main analyses utilize all data. In addition, supplementary results repeat key analyses that utilize ‘normalized peaks’ timeseries, in order to exclude the possibility that findings are confounded by differential head sizes of participants, in particular, with regard to linear speed. We extracted peaks (speed maxima) in the raw speed timeseries and normalized them by surrounding peaks in order to account for the possible variable scaling relations in speed due to head and body size (cf. ref. 45):

3 $$Normalized\,Peaks=\frac{Speed(peak)}{Speed(peak)+Speed(average\,(two\,neighboring\,peaks))}.$$

A similar analytic strategy—extracting movement data from functional or resting-state fMRI data and applying distributional analyses on the output—was used in two recent works^23, 45^.

We performed analyses (please see below) on individual participant’s data, as well as pooled data across participants of the relevant subgroups, including infants at High and Low risk for developing autism. Subgroup analyses proceeded by combining individual participant-level data, and then by performing a single distributional fit on the timeseries.

Prior to the main statistical analyses, we first established that our observed data (21,834 speed data points across all 93 rs-fMRI datasets, examined separately for linear and angular speeds) are not normally distributed using 3 tests, Kolmogorov-Smirnov, Lilliefors, and Jarque-Bera. Both linear and angular speeds, either raw or normalized versions were found to be not normally distributed (K-S, p < 0.001; Lilliefors, p = 1.0000e-03; Jarque-Bera, p = 1.0000e-03). Further, HR and LR infants’ distributions were significantly different from each other when considering the entire sample as well as infants according to each age group (1–2 and 9–10 mo-olds). Specifically, empirical cumulative distribution functions (eCDFs) were significantly different for HR and LR infants when considering all available datasets (N = 49_HR_ vs. N = 44_LR_) for both linear (two-sample K-S test, D = 0.0601, p = 6.5195e-18) and angular (two-sample K-S test, D = 0.0761, p = 1.7935e-28) speeds (eCDFs are shown in Fig. 1). Group differences were significant when considering HR and LR distributions at each age group. eCDFs of 1–2 mo-olds were significantly different between HR and LR infants (N = 28_HR_ vs. N = 28_LR_) for both linear (two-sample K-S test, D = 0.0409, p = 2.4635e-05) and angular (two-sample K-S test, D = 0.0592, p = 1.0415e-10) speeds. eCDFs of 9–10 mo-olds were significantly different between HR and LR infants (N = 21_HR_ vs. N = 16_LR_) for both linear (two-sample K-S test, D = 0.0880, p = 3.7688e-15) and angular (two-sample K-S test, D = 0.1195, p = 1.3533e-27) speeds.

#### Statistical analyses

We examined the quantitative character of subtle fluctuations of head movements using the Gamma probability distribution (PD). Gamma distribution is a two parameter family of continuous PDs, ranging from Gaussian (normally distributed, symmetrical) to Exponential (more random and noisy, heavy-tailed). Although previous work shows that parameter estimates of typically developing individuals fall closer to the Gaussian ranges relative to individuals with neurodevelopmental disorders, including ASD, there are notable exceptions, including individuals with ADHD-inattentive type who show more Gaussian signatures relative to controls^45^. The use of the Gamma parameter plane confers flexibility in the study of statistical properties of the signal since it does not require *a priori* assumptions about the underlying distribution of data.

The probability density function (PDF) of the Gamma distribution is defined as:

4 $${\rm{y}}=f(x|a,b)=\frac{1}{{b}^{a}{\rm{\Gamma }}(a)}{x}^{a-1}{e}^{\frac{-x}{b}},\,{\rm{for}}\,x > 0$$

where Γ(·) is the Gamma function (modeling sums of exponentially distributed random variables), and *a* and *b* are its *shape* and *scale* parameters, respectively. Large *a* values (presented along the x-axis) indicate that the distribution is closer to a normal (Gaussian) distribution, whereas smaller values indicate a shift toward a more Exponential distribution. The scale, *b*, parameter of the Gamma distribution (presented along the y-axis) corresponds^45^ to the Fano Factor (FF)^46^, a noise to signal measure expressed as the ratio of variance over mu: $FF=\frac{{\sigma }^{2}}{\mu }$. Higher (worse) levels of noise relative to signal on the *b* scale parameter (FF) are located towards the upper range of the y-axis. MATLAB’s fitdist function estimates 95% CIs for Gamma fits using a maximum likelihood estimation (MLE) procedure. We used functions in the Statistics and Machine Learning and Curve Fitting Toolboxes in MATLAB 8.3 (R2014a) (MathWorks, Natick, MA, USA) for all statistical analyses. We studied the power relation (f(x) = a*x^b) between individual participants’ parameter estimates of shape and scale to help visualize deviation from linearity for infants at High vs. Low risk for developing ASD. Kruskal-Wallis (a non-parametric test of analysis of variance) tested whether residuals of polyfit to individual parameter estimates were different between HR and LR infants (level of significance was set at alpha = 0.05).

In the empirically estimated parameters of Gamma PD of speeds in the 1^st^ year of life we assessed for the differential effects of High vs. Low autism risk status, first across all age groups and then separately for 1–2 mo-olds and 9–10 mo-olds. In the longitudinal subset of 22 infants (N = 11_HR_ and N = 11_LR_), we also probed the trajectories of individuals as a function of magnitude of noise-to-signal levels at 1–2 months compared to 9–10 months, for HR and LR infants. First, 1–2 mo-olds were grouped into “low” or “high” noise group using median score of their movement signatures at time point 1 (the same groupings were obtained when using either the *a* or *b* parameter estimates). Then for each risk group, we created subgroupings of these infants—now 9–10 mo-olds—as follows: HR_lownoise_, N = 5, HR_highnoise_, N = 6, LR_lownoise_, N = 6, LR_highnoise_, N = 5.

Crucially, we studied the link between stochastic signatures during the 1^st^ year of life and proximal and concurrent developmental outcomes. We asked if the signatures were ‘worse’ for infants presenting with an atypical developmental trajectory during their first 2 years of life using the Early Learning Composite (ELC) standard score on the Mullen Scales of Early Learning^47^. This composite score takes into account the child’s performance on four subscales: Expressive Language (EL), Receptive Language (RL), Visual Reception (VR), and Fine Motor (FM). For each infant, scores at multiple time points (e.g., 6, 12, and/or 18 months) were available. In this analysis, separately for each risk group, we established each individual infant’s “trajectory” and then subgrouped infants into a ‘progress’ group (i.e., those individuals with rapidly accelerating trajectories (e.g., ref. 38), consistent with more normative patterns of development (http://www.cdc.gov/ncbddd/actearly/milestones/milestones-18mo.html) through 2–3 years of age, when important milestones such as vocabulary growth reach an inflection point and a ‘stuck’ group (those who showed a flatter trajectory during the same period).

Prior to forming subgroupings, here we first searched for the presence of the expected age-related developmental improvement trajectory in these data by examining, cross-sectionally (using all available data), the scores of the entire sample (107 data points from 53 individual infants with available data) as a function of age, and then separately by risk group (HR: 60 data points from 29 infants 6 to 22 months age; LR: 47 points from 25 infants, 6 to 18 months range). (Note that we used T-scores, as raw scores were available for each of the subscales but not for Early Learning Composite (ELC) standard scores; raw scores represent values that have not been adjusted for age. Developmental trajectory fits using raw values for subscales only are listed in the Supplementary Results).

We ascertained the best model for our data using a goodness-of-fit (GoF) with the sum of the squared residual error (SSE) and a degree-of-freedom-adjusted R-square (R^2^) by considering power, f(x) = a*x^b and exponential, f(x) = a*exp(b*x) equations for both groups. R-square was used to contrast GoF between the different functions. Below we report both Power and Exponential fits across all participants for ELC standard scores, followed by Power fits only for ELC and each of the subscales (including Gross Motor (GM)) for each of the risk subgroups, as the power equation yielded better fits to these data.

Across all participants, ELC Power fit is given by f(x) = a*x^b^, with the *b* exponent representing the slope (a = 100.2 (86.61, 113.8), b = 0.01838 (−0.041 relative to LR infants (Supplementary 25, 0.078), goodness-of-fit SSE 2.08e + 04, DFE 105, R^2^: 0.003657, Adjusted R^2^: −0.005832, RMSE: 14.07); ELC Exponential fit is given by f(x) = a*exp(b*x), (a = 104.5 (97.77, 111.1), b = −4.976e-05 (−0.005786, 0.005687), goodness-of-fit SSE 2.087e + 04, DFE 105, R^2^: 2.932e-06, Adjusted R^2^: −0.009521, RMSE: 14.1).

For the **LR group**, ELC Power fit is given by f(x) = a*x^b^, with the *b* exponent representing the slope (a = 77.45 (66.53, 88.36), b = 0.148 (0.08556, 0.2104), goodness-of-fit SSE 4001, DFE 45, R^2^: 0.3341, Adjusted R^2^: 0.3193, RMSE: 9.429). EL Power fit, f(x) = a*x^b^, with the *b* exponent representing the slope (a = 37.25 (27.13, 47.37), b = 0.1321 (0.01147, 0.2528), goodness-of-fit SSE 3212, DFE 45, R^2^: 0.09515, Adjusted R^2^: 0.07504, RMSE: 8.449). RL Power fit, f(x) = a*x^b^, with the *b* exponent representing the slope (a = 33.3 (25.22, 41.38), b = 0.1956 (0.0889, 0.3022), goodness-of-fit SSE 2684, DFE 45, R^2^: 0.2243, Adjusted R^2^: 0.2071, RMSE: 7.724). VR Power fit, f(x) = a*x^b^, with the *b* exponent representing the slope (a = 41.65 (31.47, 51.83), b = 0.1384 (0.02999, 0.2467), goodness-of-fit SSE 3336, DFE 45, R^2^: 0.1277, Adjusted R^2^: 0.1083, RMSE: 8.61). GM Power fit, f(x) = a*x^b^, with the *b* exponent representing the slope (a = 50.96 (34.42, 67.49), b = −0.01071 (−0.158, 0.1365), goodness-of-fit SSE 4626, DFE 45, R^2^: 0.0004699, Adjusted R^2^: −0.02174, RMSE: 10.14). FM Power fit, f(x) = a*x^b^, with the *b* exponent representing the slope (a = 42.22 (30.77, 53.68), b = 0.1352 (0.01479, 0.2557), goodness-of-fit SSE 4174, DFE 45, R^2^: 0.1051, Adjusted R^2^: 0.08521, RMSE: 9.631).

For the **HR group**, ELC Power fit is given by f(x) = a*x^b^, with the *b* exponent representing the slope (a = 116.7 (93.13, 140.2), b = −0.0575 (−0.1454, 0.03042), goodness-of-fit SSE 1.389e + 04, DFE 58, R^2^: 0.02999, Adjusted R^2^: 0.01326, RMSE: 15.47). EL Power fit, f(x) = a*x^b^, with the *b* exponent representing the slope (a = 48.3 (33.16, 63.44), b = −0.009971 (−0.1455, 0.1255), goodness-of-fit SSE 7067, DFE 58, R^2^: 0.0003837, Adjusted R^2^: −0.01685, RMSE: 11.04). RL Power fit, f(x) = a*x^b^, with the *b* exponent representing the slope (a = 63.17 (46.51, 79.83), b = −0.1133 (−0.2293, 0.002738), goodness-of-fit SSE 5451, DFE 58, R^2^: 0.06404, Adjusted R^2^: 0.0479, RMSE: 9.695). VR Power fit, f(x) = a*x^b^, with the *b* exponent representing the slope (a = 61.71 (45.29, 78.13), b = −0.06077 (−0.1768, 0.05523), goodness-of-fit SSE 6661, DFE 58, R^2^: 0.01964, Adjusted R^2^: 0.002736, RMSE: 10.72). GM Power fit, f(x) = a*x^b^, with the *b* exponent representing the slope (a = 53.02 (40.25, 65.78), b = −0.02578 (−0.1302, 0.07859), goodness-of-fit SSE 4690, DFE 58, R^2^: 0.004176, Adjusted R^2^: −0.01299, RMSE: 8.993). FM Power fit, f(x) = a*x^b^, with the *b* exponent representing the slope (a = 64.47 (48.07, 80.88), b = −0.07575 (−0.187, 0.03546), goodness-of-fit SSE 6228, DFE 58, R^2^: 0.03328, Adjusted R^2^: 0.01662, RMSE: 10.36).

As expected, LR infants and toddlers have a higher (steeper) *b* exponent (slope) with 95% CIs that do not overlap with 95% CIs of HR infants on the standard Mullen Early Learning Composite score, ELC (LR *b* = 0.148 (0.08556, 0.2104) vs. HR *b* = −0.0575 (−0.1454, 0.03042)) (Supplementary Figure 5, top panel), with a consistent pattern for each of the subscales, as well as when using raw scores (Supplementary Results), indicating a more rapid rise in developmental trajectory as a function of age in the Low Risk group. In addition, note that 95% CIs for *b* overlap 0 for the HR group, suggesting the presence of individual differences in overall developmental progress.

An atypical behaviorally-defined trajectory in the HR relative to the LR group in our data is also supported by trajectories on the Vineland Adaptive Behavior Composite (ABC) standard scores^49^ available for a total of 43 data points (from 29 infants, over the range of 12 to 30 months). 15 HR infants provided 22 data points and 14 LR infants provided 21 data points. The LR group's ABC Power fit is given by f(x) = a*x^b^, with the *b* exponent representing the slope (a = 74.03 (44.77, 103.3), b = 0.1165 (−0.02472, 0.2577), goodness-of-fit SSE 1758, DFE 19, R^2^: 0.1344, Adjusted R^2^: 0.08883, RMSE: 9.62). The HR group's ABC Power fit is given by f(x) = a*x^b^, with the *b* exponent representing the slope (a = 137.5 (42.65, 232.4), b = −0.1375 (−0.3866, 0.1116), goodness-of-fit SSE 4543, DFE 20, R^2^: 0.06647, Adjusted R^2^: 0.0198, RMSE: 15.07) (Supplementary Figure 5, bottom panel). Our data thus show atypically *shallower* slopes on behavioral outcomes as a function of age for HR infants relative to LR infants.

#### External validation of HR developmental pattern on the Mullen and/or Vineland in 1,445 HR and LR infants

We used external validation to probe the developmental pattern observed above, using available Mullen and/or Vineland scores assessed at 2 to 7 years, from 4 recent representative High Risk infant-siblings studies with known ASD outcomes for HR infants from both US and UK samples^25–28^. We analyzed data from 1,445 infants, ~945 HR and ~500 LR, computing 95% Confidence Intervals around the mean, using standard deviation (*s*), mean of sample ($\bar{x}$), and sample size (*n*) values and the following formula:

5 $$\bar{x}\pm 1.96\ast \frac{s}{\sqrt{n}}$$

(These values are available in the Extended Data Table 1 in ref. 28; Table S1 and Table 2 in ref. 25; Supplementary Table 4 in ref. 26 and Table on p. 11 of the Supplementary Information in ref. 27). (Note that raw values for LR infants were not available for ref. 27; raw values for HR ASD-positive infants were not available for ref. 26). These data from 1,445 infants show *lower* scores on either Mullen and/or Vineland inventories in HR infants, whether or not the infant eventually received ASD diagnosis (Supplementary Figure 6).

Specifically, developmental trajectories of HR infants who were “ASD-negative” (no clinical ASD diagnosis) were not only—conservatively speaking—*no better than those of LR infants*, with no overlap in 95% CIs of the mean on either Mullen or Vineland instruments, but moreover, 95% CIs of HR ASD-negative infants *overlapped* with 95% CIs of HR ASD-positive infants in some samples. Note that the ordering of the means (LR showed the highest scores, followed by HR ASD-negative and then HR ASD-positive infants) is consistent for each study, for relatively modest-sized samples (the UK BASIS sample^25^ comprised ~N = 76, with N = 37 LR, N = 24 HR_ASDneg_, N = 15 HR_ASDpos_ at the 7-year follow-up assessment) and larger samples (US IBIS sample^28^ comprised ~N = 435, with N = 117 LR, N = 248 HR_ASDneg_, and N = 70 HR_ASDpos_).

### Recurrence rates

Ozonoff and colleagues^48^ noted that the recurrence risk for HR siblings is around 18.7%, ranging between 13.34 and 25.5 (95% CIs), and that the “true recurrence rate may in fact be higher than that reported here”^48^ due to emergence of milder forms of atypical functioning as children grow. Based on our analyses above, Hazlett and colleagues’^28^ rate (at 2 years) is around 22% (N = 70 ASDpos, N = 248 ASDneg), while Shepard and colleagues’^25^ recurrence rate at a 7-year follow-up is around 38% (N = 15 ASDpos, N = 24 ASDneg).

In addition to the question of whether or not a HR sibling may receive an ASD diagnosis, investigators have pointed out atypical functioning in a substantial proportion of HR ASD-negative siblings. For example, Shepard and colleagues^25^ reported that 20 to 30% of HR non-ASD siblings (twice the number of LR siblings) received scores on the Connors scale (assessing behaviors including ‘inattention’) above clinical threshold relative to LR infants, as well as elevated anxiety scores relative to LR infants. Charman and colleagues^26^ studied only HR ASD-negative infants relative to LR infants (no HR ASD-positive comparison), and found rates of mild-to-moderate developmental delay 3 times higher in HR ASD-negative infants relative to LR infants, as well as elevated ASD symptoms. In the current work, we use these values as a guide to estimate that the number of HR infants who may receive an ASD diagnosis in childhood would be between ~22% to 38%, with an additional 20 to 30% presenting with atypical outcomes. Thus, based on estimates from these observational instruments, up to ~68% of HR infants are expected to have atypical development later in life. Here we used a data-driven procedure to subgroup infants according to their developmental trajectory in order to predict atypical developmental outcomes using quantitative measures after birth.

### Subgroupings by developmental trajectory using power fit exponents

Subgroupings of individuals with at least 2 time points on Mullen were created as follows. We used T-scores to create individual fits, and then used exponents of the power fits to rank-order individuals, with *higher* exponents designating more rapid (higher or faster rise of the function) progress of the individual over time. For each risk group separately, individuals were median-ranked by their exponent, according to whether they were ‘stuck’ (their exponent was lower) or whether they made ‘progress’ (exponent higher) over the data points tested. (In pilot work we also created fits by normalizing raw scores between 0 and 1 (normMUscore = (Score_RAW_-WithinGroupScore_MIN_/WithinGroupScore_MAX_-WithinGroupScore_MIN_)) and used these normalized scores to create individual fits; we obtained similar subgroupings as when using T-scores).

All individuals used in the groupings had at least 1 data point less than 9–10 months (at 6 months), and at least 1 data point greater than 9–10 months (at 12 months), ensuring an overlap between the trajectory on these Mullen records and the time of the scan at 9–10 months.

A total of 28 HR and 28 LR infants were scanned at 1–2 months, with the following Ns that specifically had at least 2 time points on the Mullen (N = 13_HR_, N = 15_LR_).The subgroupings were comprised as follows: for Mullen ELC, HR_stuck_: N = 6_ELC_, HR_progress_: N = 7_ELC_, LR_stuck_: N = 7_ELC_, LR_progress_: N = 8_ELC_.

A total of 21 HR and 16 LR infants were scanned at 9–10 months, with the following Ns that specifically had at least 2 time points on the Mullen (N = 14_HR_, N = 13_LR_). The subgroupings were comprised as follows: for Mullen ELC, HR_stuck_: N = 7_ELC_, HR_progress_: N = 7_ELC_, LR_stuck_: N = 7_ELC_, LR_progress_: N = 6_ELC_.

Post-hoc, we also used available Vineland ABC scores^49^ obtained around 12 months, grouping infants into those with lower vs. those with higher scores to test the link to movement signatures at 9–10 months. (Note that few infants with imaging data had scores available on the Vineland scale). The subgroupings were comprised as follows: for Vineland ABC, HR_low_: N = 3_ABC_, HR_high_: N = 3_ABC_, LR_low_: N = 3_ABC_, LR_high_: N = 4_ABC._

### Subgroupings by father’s age: probing biological origins of infant movement signatures

For a subset of infants for whom their father’s age at the time of conception was available, we formed subgroupings as follows. For each risk group, Father’s Ages (FA) were ranked and split into 2 subgroupings, those with above and below the median age. Data are presented separately for 1–2 and 9–10 mo-olds. The following are Ns for 1–2 mo-olds: HR_FAHigh_, N = 6, HR_FALow_, N = 5, LR_FAHigh_, N = 4, LR_FALow_, N = 3 and for 9–10 mo-olds: HR_FAHigh_, N = 6, HR_FALow_, N = 7, LR_FAHigh_, N = 3, LR_FALow_, N = 2. The following are mean, std, and range for Father’s age subgroupings for 1–2 mo-olds: HR_FAHigh_ mean, std: (41.5 +/− 1.76); range, min-max: 39–43; HR_FALow_ mean, std: (34.6 +/− 3.51); range, min-max: 29–38; LR_FAHigh_ mean, std: (39.25 +/− 1.5); range, min-max: 38–41; LR_FALow_ mean, std: (31 +/− 4); range, min-max: 27–35 and for 9–10 mo-olds: HR_FAHigh_ mean, std: (41.83 +/− 2.23); range, min-max: 39–45; HR_FALow_ mean, std: (34.71 +/− 2.14); range, min-max: 32–38; LR_FAHigh_ mean, std: (40.66 +/− 2.52); range, min-max: 38–43; LR_FALow_ mean, std: (36.5 +/− 2.12); range, min-max: 35–38. For 1–2 mo-olds, the difference in mean father’s age between HR subgroups is 6.9 and between LR subgroups: 8.25. For 9–10 mo-olds, the difference in mean father’s age between HR subgroups is 7.12 and between LR subgroups: 4.16. (Analyses focus on resting-state data for 1–2 and 9–10 mo-olds; too few infants with a resting-state scan at 9–10 months who also had a native language scan, had available father’s ages. Thus, analyses reporting movements during a native language scan as a function of father’s age focus on 1–2 mo-olds).

In summary, for each subgroup considered we analyzed statistical character of subtle fluctuations in speeds of spontaneous head movements on the Gamma parameter plane. We characterize the contribution of biological (i.e., autism risk status) and experimental (i.e., native language listening vs. resting or sleeping) factors in movement signatures in human infants. In particular, we use a data-driven procedure to independently define developmental trajectory groupings and, for each subgrouping, we derive parameter estimates of speeds of head movements on the Gamma parameter plane that we quantitatively characterize using power-law (and exponential) relations. Comparing movement signatures during a native language listening task and those during sleep, we searched for a developmentally-driven early origin of atypical development in HR infants. Non-parametric Wilcoxon signed rank test compared fits for N = 27 1–2 mo-old HR infants with both native language listening and resting-state fMRI scans, and for N = 28 1–2 mo-old LR infants with both types of scans. Finally, in an exploratory analysis, infants with available data were subgrouped by higher or lower father’s ages in order to probe prenatal origins of these signatures.

## Electronic supplementary material

Supplementary Materials
