# Supplementary material for: Inflexible neurobiological signatures precede atypical development in infants at high risk for autism
Source: Sci Rep. 2017 Sep 12;7:11285. doi: 10.1038/s41598-017-09028-0 (PMC5595985; doi:10.1038/s41598-017-09028-0)
Supplement: Supplementary file 1 — Supplementary Materials [file 41598_2017_9028_MOESM1_ESM.pdf]

SUPPLEMENTARY MATERIAL

Inflexible neurobiological signatures precede atypical development in infants at high risk for autism

KRISTINA DENISOVA[a,b,c *] **AND GUIHU ZHAO**[b]

[a]Sackler Institute for Developmental Psychobiology, Columbia University College of Physicians and Surgeons, New York, NY 10032, USA

[b]Department of Psychiatry, Columbia University College of Physicians and Surgeons, New York, NY 10032, USA;

[c]Division of Developmental Neuroscience, New York State Psychiatric Institute, New York, NY 10032, USA

***Corresponding author**: Kristina Denisova, Ph.D., Division of Developmental Neuroscience, Department of Psychiatry, Columbia University, 1051 Riverside Drive, Unit 40, New York, NY 10032. Email: kd2401@cumc.columbia.edu

SUPPLEMENTARY TABLES

		Coefficients (with 95 % confidence intervals)		Goodness-of-Fit				
		a	b	SSE	RSquare	DFE	AdjRSquare	RMSE
Entire sample (N=93)	Linear	0.07467 (0.05624, 0.0931)	-1.97 (-2.341, -1.6)	0.2727	0.6261	91	0.6220	0.0547
	Angular	0.03427 (0.01589, 0.05265)	-3.24 (-3.893, -2.588)	0.6447	0.7162	91	0.7131	0.0842
HR only (N=49)	Linear	0.0857 (0.05473, 0.1167)	-1.873 (-2.398, -1.349)	0.2362	0.6477	47	0.6402	0.0709
	Angular	0.04617 (0.01428, 0.07807)	-2.995 (-3.823, -2.167)	0.5476	0.7292	47	0.7234	0.1079
LR only (N=44)	Linear	0.04946 (0.04141, 0.0575)	-1.318 (-1.647, -0.9897)	0.0107	0.6051	42	0.5957	0.0160
	Angular	0.03644 (0.02616, 0.04673)	-2.495 (-2.907, -2.082)	0.0160	0.8949	42	0.8924	0.0195
1-2 HR mo-olds (N=28)	Linear	0.0769 (0.0387, 0.1151)	-1.464 (-2.272, -0.655)	0.1497	0.3910	26	0.3676	0.0759
	Angular	0.06715 (0.02622, 0.1081)	-1.617 (-2.643, -0.5908)	0.1814	0.4096	26	0.3869	0.0835
1-2 LR mo-olds (N=28)	Linear	0.04715 (0.0364, 0.0579)	-1.224 (-1.694, -0.7534)	0.0090	0.5269	26	0.5087	0.0187
	Angular	0.02967 (0.01494, 0.04439)	-2.917 (-3.764, -2.07)	0.0142	0.8361	26	0.8298	0.0234
9-10 HR mo-olds (N=21)	Linear	0.0669 (0.04457, 0.08923)	-2.649 (-3.106, -2.192)	0.0134	0.9682	19	0.9666	0.0265
	Angular	0.06028 (0.01089, 0.1097)	-2.923 (-3.857, -1.99)	0.1314	0.9212	19	0.9170	0.0832
9-10 LR mo-olds (N=16)	Linear	0.06439 (0.05631, 0.07247)	-1.838 (-2.144, -1.531)	0.0005	0.9220	14	0.9164	0.0064
	Angular	0.04385 (0.03237, 0.05534)	-2.223 (-2.551, -1.896)	0.0011	0.9836	14	0.9824	0.0087

Supplementary Table 1. The results of the power curve fits of the form f(x)=a*x^b, fitted to the Gamma shape and scale parameters of head movement fluctuations, for raw angular and linear speeds (N=93 datasets), separately for High Risk ($N=49_{HR}$) and Low Risk ($N=44_{LR}$) infants across age, as well as for HR and LR infants in the two age groups, 1-2 mo-olds ($N=28_{HR}$, $N=28_{LR}$) and 9-10 mo-olds ($N=21_{HR}$, $N=16_{LR}$). The RMSE (root mean squared error) is lower for the LR group, indicating a better fit, for both angular and linear speeds. Note that LR infants have consistently lower RMSE values for each of the age groups considered. Specifically, both LR 1-2 and LR 9-10 mo-olds have lower RMSE relative to HR 1-2 and 9-10 mo-olds.

		Coefficients (with 95 % confidence intervals)		Goodness-of-Fit				
		a	b	SSE	RSquare	DFE	AdjRSquare	RMSE
Power Fit: MU ELC	Linear	0.003723 (0.0004588,0.006987)	-3.759 (-4.506, -3.013)	3.0229e-05	.9995	2	.9993	.0039
	Angular	0.009319 (-0.0123, 0.03094)	-4.139 (-6.69, -1.588)	5.7159e-04	.9948	2	.9922	.0169
Exponential Fit: MU ELC	Linear	4.224 (0.6311, 7.816)	-8.457 (-11.18, -5.73)	7.4832e-05	.9989	2	.9983	.0061
	Angular	11.89 (-20.89, 44.67)	-8.395 (-15.2, -1.59)	8.5441e-04	.9923	2	.9884	.0207

Supplementary Table 2. The results of the power curve fits of the form f(x)=a*x^b as well as of the exponential fits of the form f(x) = a*exp(b*x) fitted to the Gamma shape and scale parameters of head movement fluctuations at **9-10 months** (raw linear and angular speed), for infants subgrouped by the rapidity of their progress over time on Mullen (MU) Scales of Early Learning trajectories. *Note*. ELC: Early Learning Composite score. Both equations fit the data well. Power fits yield higher RSquare and lower RMSE (root mean square error), indicating a slightly better fit for these data.

		Coefficients (with 95 % confidence intervals)		Goodness-of-Fit				
		a	b	SSE	RSquare	DFE	AdjRSquare	RMSE
Power Fit: MU ELC	Linear	0.003851 (-0.001968, 0.009671)	-3.242 (-4.761, -1.723)	5.0243e-05	.9919	2	.9879	.0050
	Angular	0.02781 (0.01997, 0.03565)	-2.468 (-2.852, -2.085)	2.1809e-05	.9988	2	.9982	.0033
Exponential Fit: MU ELC	Linear	1.304 (-1.549, 4.156)	-6.988 (-12.76, -1.215)	1.3118e-04	.9789	2	.9684	.0081
	Angular	1.144 (0.1513, 2.137)	-3.922 (-5.619, -2.225)	1.5153e-04	.9917	2	.9876	.0087

Supplementary Table 3. The results of the power curve fits of the form f(x)=a*x^b as well as of the exponential fits of the form f(x) = a*exp(b*x) fitted to the Gamma shape and scale parameters of head movement fluctuations at **1-2 months** (raw linear and angular speed), for infants subgrouped by the rapidity of their progress over time on Mullen (MU) Scales of Early Learning trajectories. *Note*. ELC: Early Learning Composite score. Both equations fit the data well. Power fits yield higher RSquare and lower RMSE (root mean square error), indicating a slightly better fit for these data.

SUPPLEMENTARY RESULTS

Power fits for High and Low Risk groups using raw scores

Fits presented for each of the five Mullen Early Learning subscales: Expressive Language (EL), Receptive Language (RL), Visual Reception (VR), Gross Motor (GM), and Fine Motor (FM). For the Low Risk (LR) group, for EL, Power fit is given by $f(x)=a*x^b$, with the *b* exponent representing the slope (a= 1.002 (0.7088, 1.294), b= 1.018 (0.9021, 1.133), goodness-of-fit SSE 111.6, DFE 45, R^2: 0.8838, Adjusted R^2: 0.8813, RMSE: 1.575). RL: Power fit $f(x)=a*x^b$, with the *b* exponent representing the slope (a= 0.9627 (0.7178, 1.208), b= 1.086 (0.986, 1.186), goodness-of-fit SSE 104, DFE 45, R^2: 0.9213, Adjusted R^2: 0.9195, RMSE: 1.52). VR: Power fit $f(x)=a*x^b$, with the *b* exponent representing the slope (a= 1.752 (1.328, 2.175), b= 0.9108 (0.8142, 1.008), goodness-of-fit SSE 149.1, DFE 45, R^2: 0.8974, Adjusted R^2: 0.8951, RMSE: 1.821). GM: Power fit $f(x)=a*x^b$, with the *b* exponent representing the slope (a= 2.092 (1.493, 2.691), b= 0.8113 (0.6955, 0.9272), goodness-of-fit SSE 197.2, DFE 45, R^2: 0.8216, Adjusted R^2: 0.8176, RMSE: 2.093). FM: Power fit $f(x)=a*x^b$, with the *b* exponent representing the slope (a= 2.036 (1.541, 2.531), b= 0.8297 (0.7315, 0.9278), goodness-of-fit SSE 145.3, DFE 45, R^2: 0.8774, Adjusted R^2: 0.8747, RMSE: 1.797).

For the High Risk (HR) group, for EL, Power fit is given by $f(x)=a*x^b$, with the *b* exponent representing the slope (a= 1.437 (0.8524, 2.022), b= 0.8374 (0.6816, 0.9932), goodness-of-fit SSE 388.8, DFE 58, R^2: 0.7079, Adjusted R^2: 0.7029, RMSE: 2.589). RL: Power fit $f(x)=a*x^b$, with the *b* exponent representing the slope (a= 1.908 (1.363, 2.453), b= 0.7728 (0.6627, 0.883), goodness-of-fit SSE 256.6, DFE 58, R^2: 0.7999, Adjusted R^2: 0.7964, RMSE: 2.103). VR: Power fit, $f(x)=a*x^b$, with the *b* exponent representing the slope (a= 2.48 (1.828, 3.132), b= 0.741 (0.6393, 0.8427), goodness-of-fit SSE 320.5, DFE 58, R^2: 0.8133, Adjusted R^2: 0.8101, RMSE: 2.351). GM: Power fit $f(x)=a*x^b$, with the *b* exponent representing the slope (a= 2.151 (1.683, 2.619), b= 0.7987 (0.7151, 0.8823), goodness-of-fit SSE 210.9, DFE 58, R^2: 0.8799, Adjusted R^2: 0.8779, RMSE: 1.907). FM: Power fit, $f(x)=a*x^b$, with the *b* exponent representing the slope (a= 2.743 (2.061, 3.424), b= 0.6865 (0.5897, 0.7832), goodness-of-fit SSE 278.1, DFE 58, R^2: 0.8044, Adjusted R^2: 0.801, RMSE: 2.19).

Consistent with the results presented in the Main text using T-scores, the b-slope is consistently higher (steeper) for the LR group.

SUPPLEMENTARY FIGURES

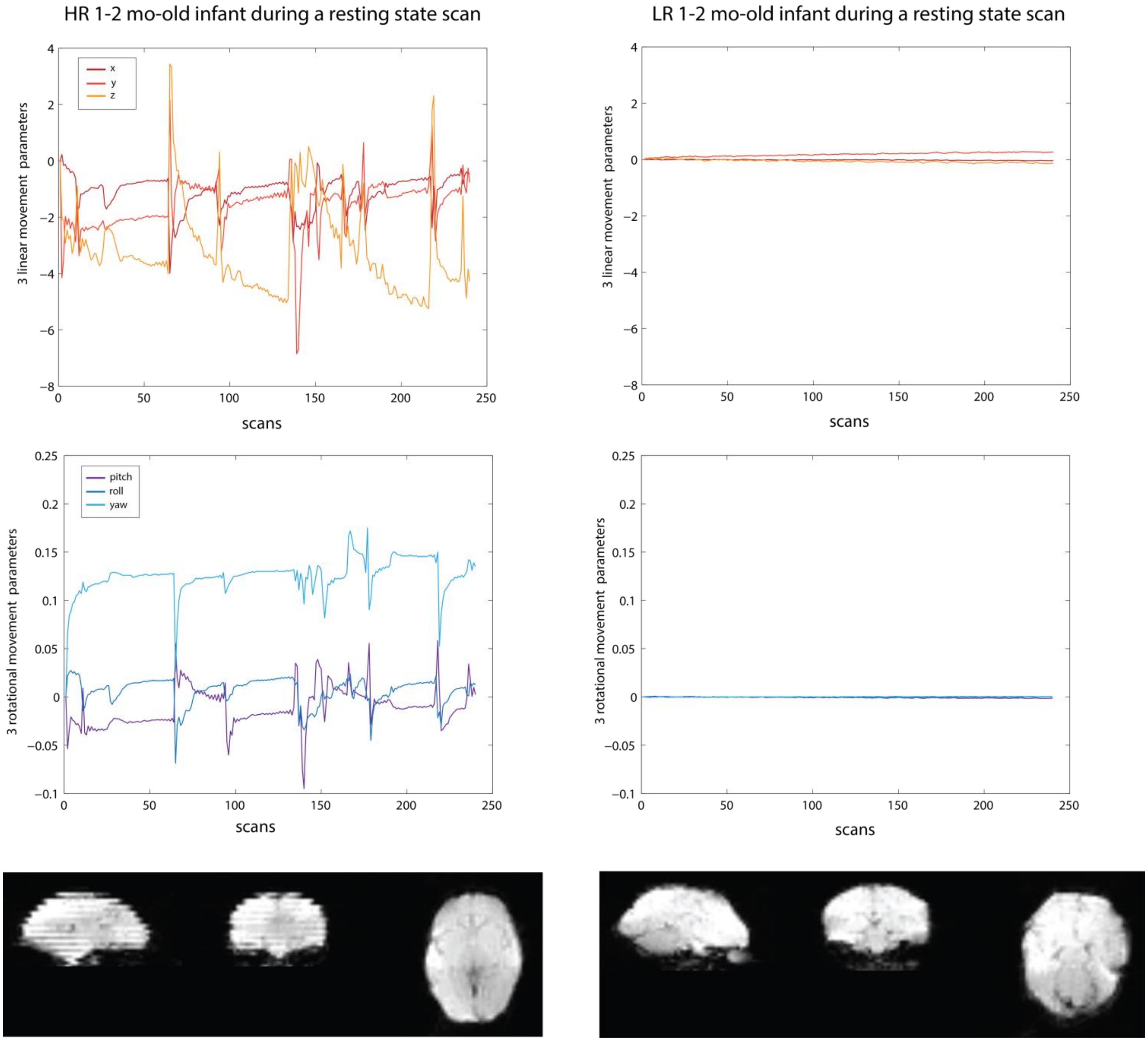

Supplementary Figure 1. Sample datasets from two infants in this study, a 1-2 mo-old HR and a 1-2 mo-old LR infant, during a resting-state scan. The top rows plot 6 movement parameter output from SPM, separately for linear (mm) and rotational (radians) values. The raw values are greater in magnitude during the entire scan for the HR infant. The bottom row shows each infant's EPI image in sagittal, coronal, and axial views displayed using MRIcron (http://people.cas.sc.edu/rorden/mricron/index.html). Note the difference in quality between the images of the two infants; the image data of the HR infant (who had greater movement during the scan) reveals visible striped lines (artefacts). (Note. Such "striping" artefacts can stem from subject movement during acquisition of brain slices in an interleaved manner; http://imaging.mrc-cbu.cam.ac.uk/imaging/CommonArtefacts).

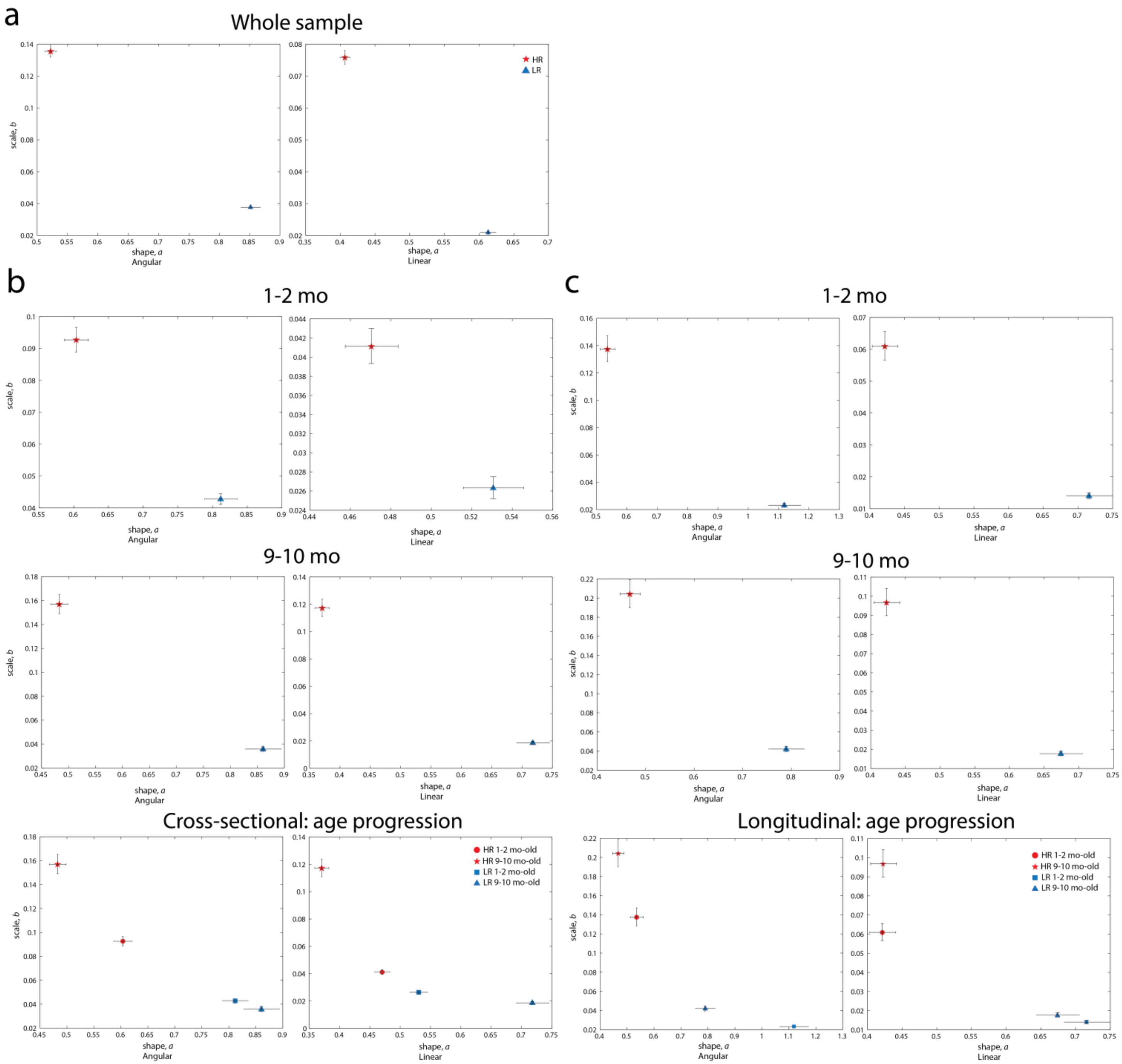

Supplementary Figure 2. Parameter estimates on the Gamma plane for subgroups of infants across all datasets and time points, cross-sectionally and longitudinally, for angular and linear speeds. (a) Parameter estimates on the Gamma plane for High and Low risk infants' distributions (total N=93: $N=49_{HR}$ and $N=44_{LR}$). (b) High and Low risk infants' cross-sectional data shown separately by age subgroups: for 1-2 month-olds ($N=28_{HR}$ and $N=28_{LR}$) and for 9-10 month olds ($N=21_{HR}$ and $N=16_{LR}$). The bottom panel presents the same data as the top panels from different age subgroups on the same plot, for angular and linear speeds. (c) High and Low risk infants' longitudinal subset data (N=22) are shown by age subgroups: for 1-2 month-olds ($N=11_{HR}$ and $N=11_{LR}$) and for 9-10 month olds ($N=11_{HR}$ and $N=11_{LR}$). The bottom panel presents the same data as the top panels, presenting data from different age subgroups on the same plot for angular and linear speeds. Regardless of the age of the infant, HR infants' parameter estimates are consistently located towards higher noise-to-signal levels (scale, *b* parameter) and towards the left on the x-axis, away from the more normative, Gaussian shape (shape, *a* parameter). Error bars denote 95% CIs.

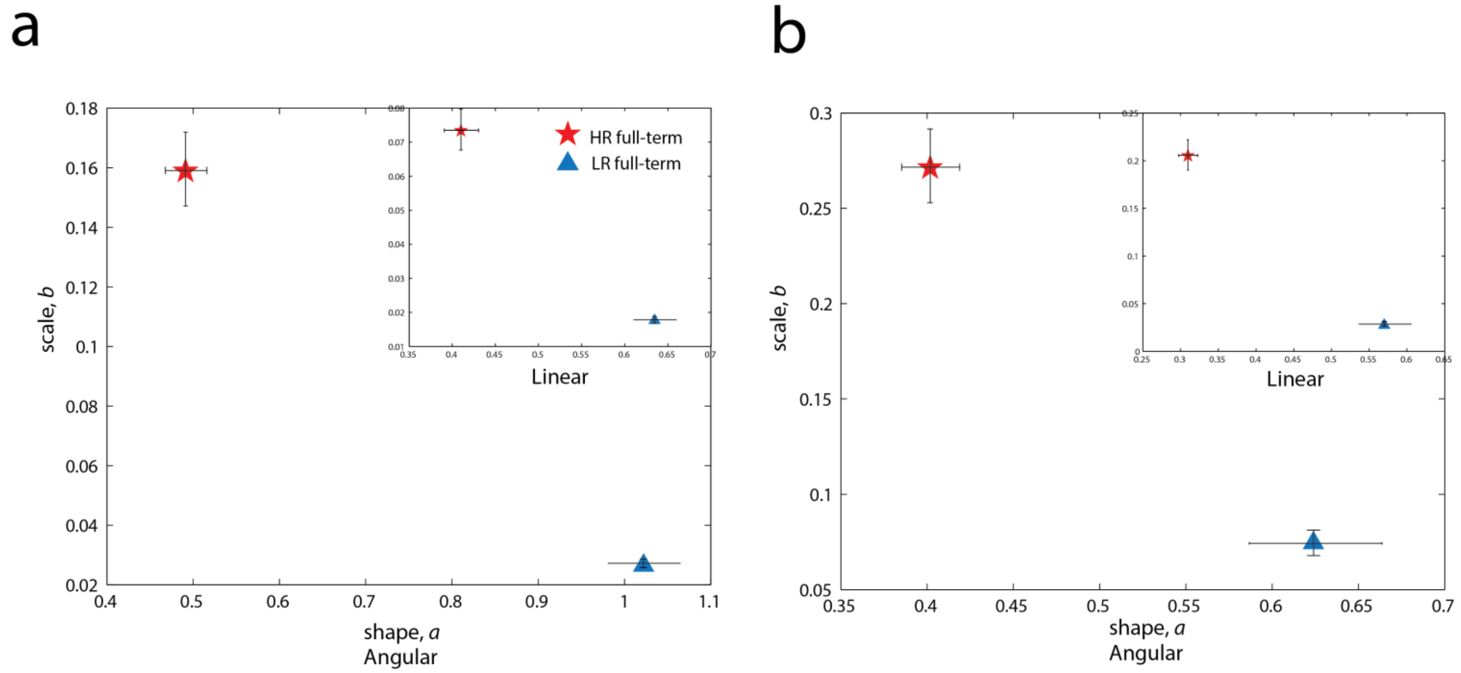

Supplementary Figure 3. Inclusion criteria at the ACE UCLA site included full-term birth. Here we confirm the main finding of our study: HR infants, specifically those with known full-term status, have increased noise-to-signal levels and decreased symmetry relative to full-term LR infants. (a) presents data for 1-2 mo-olds and (b) presents data for 9-10 mo-olds. *Note*. Data presented includes some subjects who were tested longitudinally. Total N for 1-2 mo-olds, N=24: $N=9_{HR}$, $N=15_{LR}$. Total N for 9-10 mo-olds, N=18: $N=12_{HR}$, $N=6_{LR}$. Error bars denote 95% CIs.

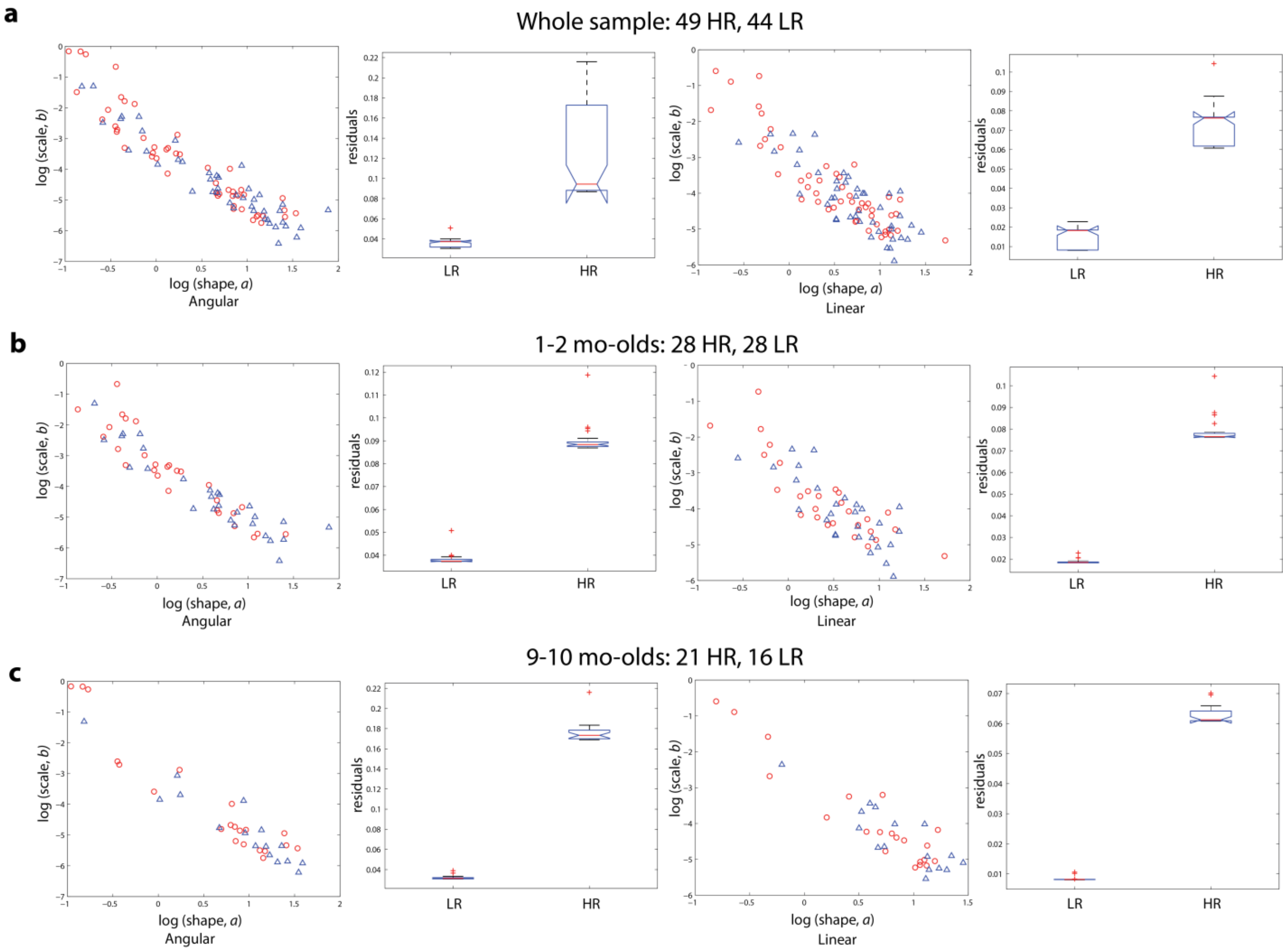

Supplementary Figure 4. Individual parameter estimates on the Gamma plane for all High and Low risk infants and residual values denoting deviation from the *a* vs *b* linear relation. Noise-to-signal levels (scale, *b* parameter; y-axis) and randomness (shape, *a* parameter; x-axis) in individual infants' spontaneous head fluctuations on the Gamma plane. Data are presented on log axes to demonstrate linearity. Lower values on the x-axis correspond to higher values on the y-axis. Data are shown for High Risk (red circles) and Low Risk (blue triangles) infants for angular and linear speed. (a) shows the entire sample (N=93 across all time points, including N=22 infants tested longitudinally), (b) shows data only for 1-2 mo-old ($N=28_{HR}$, $N=28_{LR}$) infants, and (c) shows data only for 9-10 mo-old ($N=21_{HR}$, $N=16_{LR}$) infants. For each a-c, and for angular and linear speeds, Kruskal-Wallis rank-order test on residuals (Whisker plots on the right-hand side) shows significant group differences, with HR infants showing greater deviation from linearity for angular and linear speed relative to LR infants across the entire sample, as well as separately for 1-2 mo-old and 9-10 mo-old age groups (all $p<0.001$).

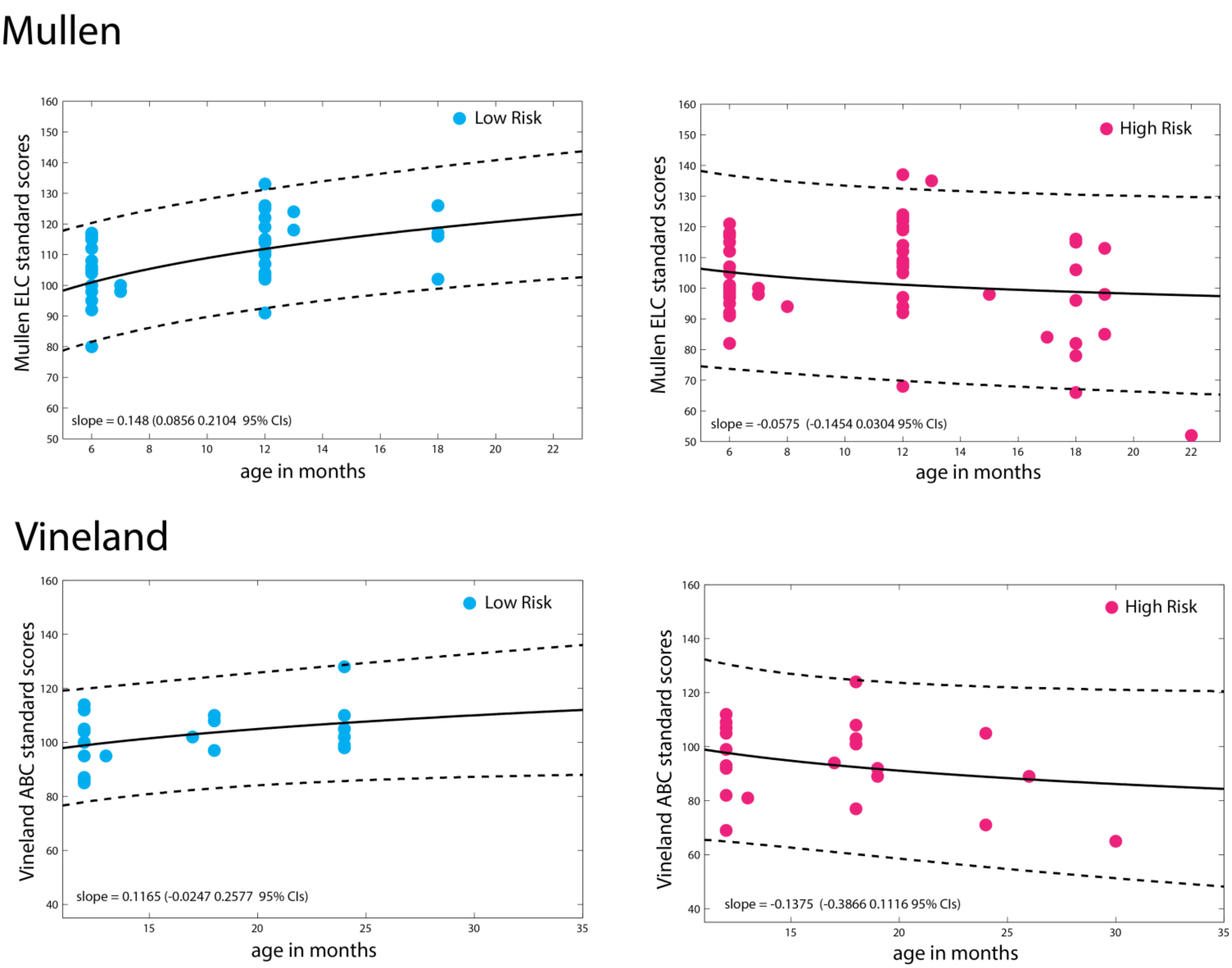

Supplementary Figure 5. Group-level developmental trajectories on the Mullen (ELC) and Vineland (ABC) for Low Risk and High Risk infants. The top row shows the Mullen ELC while the bottom row shows Vineland ABC scores. For the Mullen, in the LR group, 47 data points are contributed from 25 unique infants, and for the HR group, 60 data points are contributed by 29 infants. For the Vineland, in the LR group, 21 data points are contributed from 14 unique infants, and for the HR group, 22 data points are contributed by 15 infants. For each, slopes with 95% Confidence Intervals (CIs) are shown. Note that unlike positive slopes of the LR group, the slopes of the HR group are negative, with non-overlapping 95% CIs relative to the HR group on the Mullen. ELC: Early Learning Composite; ABC: Adaptive Behavior Composite.

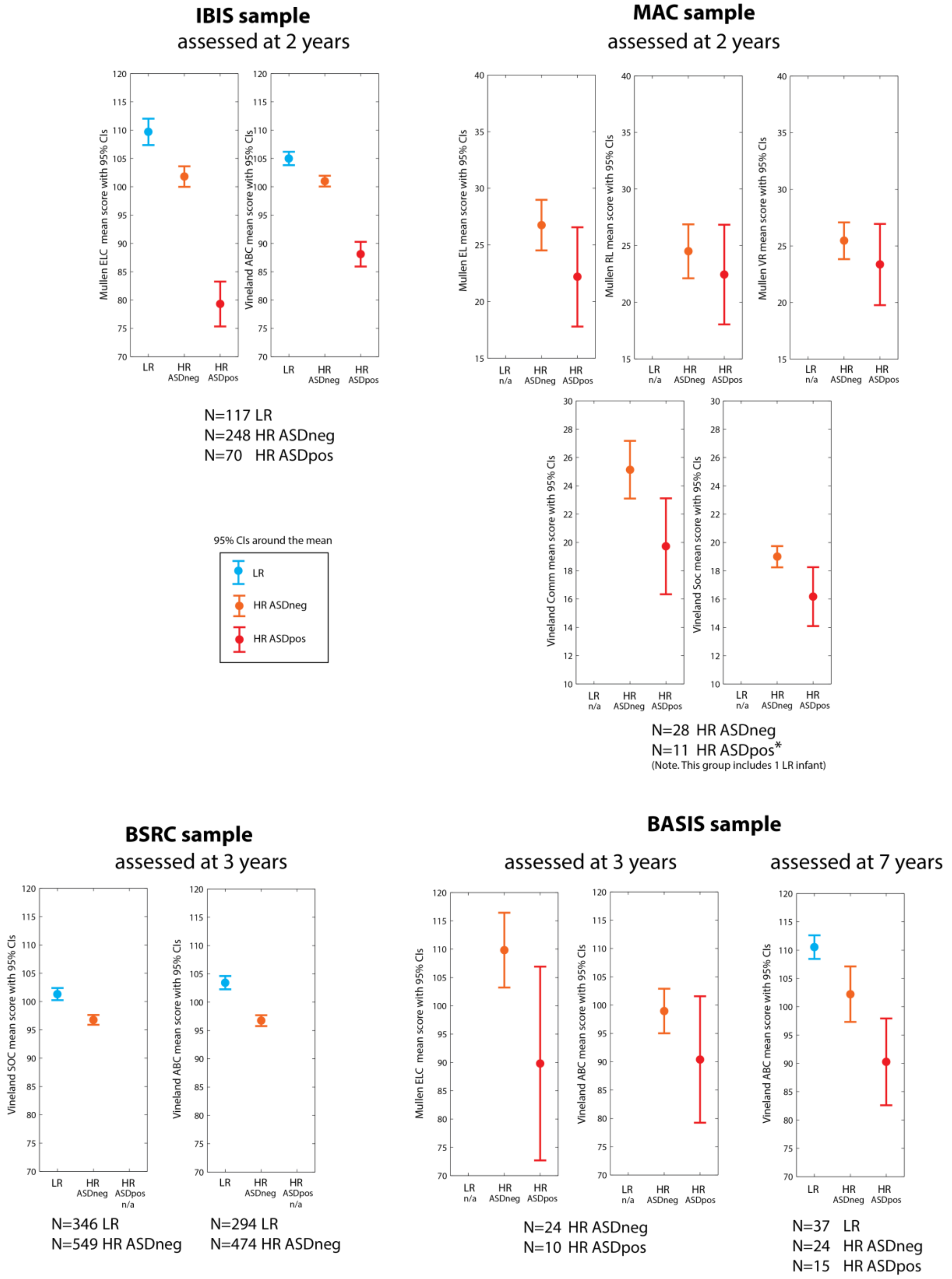

Supplementary Figure 6. External validation of developmental patterns on the Mullen and Vineland observational instruments in HR infants with known ASD diagnostic outcomes. Analyses using published data from a total of 1,445 infants (~N=500 LR and ~N=945 HR) from 4 representative infant-sibling studies: IBIS US sample (Hazlett et al., 2017; data points plotted comprised N=435), MAC US sample (Jones & Klin 2013; data points plotted comprised N=39. *Available values in Jones and Klin 2013 for the ASDpos subgroup combined 10 HR infants and 1 LR infant who received an ASD diagnosis; here all 11 subjects are represented as the "HR ASD pos" subgroup), BSRC US, UK, Israel, and Canada sample (Charman et al., 2017; the total Ns are from the Vineland SOC, Socialization subscale assessment, N=895), and BASIS UK sample (Shepard et al., 2017; the total Ns for this site are from the 7-year assessment, N=76). HR ASD-negative infants' means are lower, and 95% CIs of the mean (either on the Mullen ELC or subscales, or Vineland ABC or subscales, depending on available values) for HR ASD-negative infants do not overlap with 95% CIs of LR infants. In addition, 95% CIs of HR ASD-negative and HR ASD-positive infants do overlap, with the exception of the IBIS sample. These atypical developmental patterns (namely, the ordering of the means for LR, HR ASD-negative, and HR ASD-positive infants) are consistent across all 4 sites, and are consistent with data from the main analyses in the current study. Error bars denote 95% CIs.

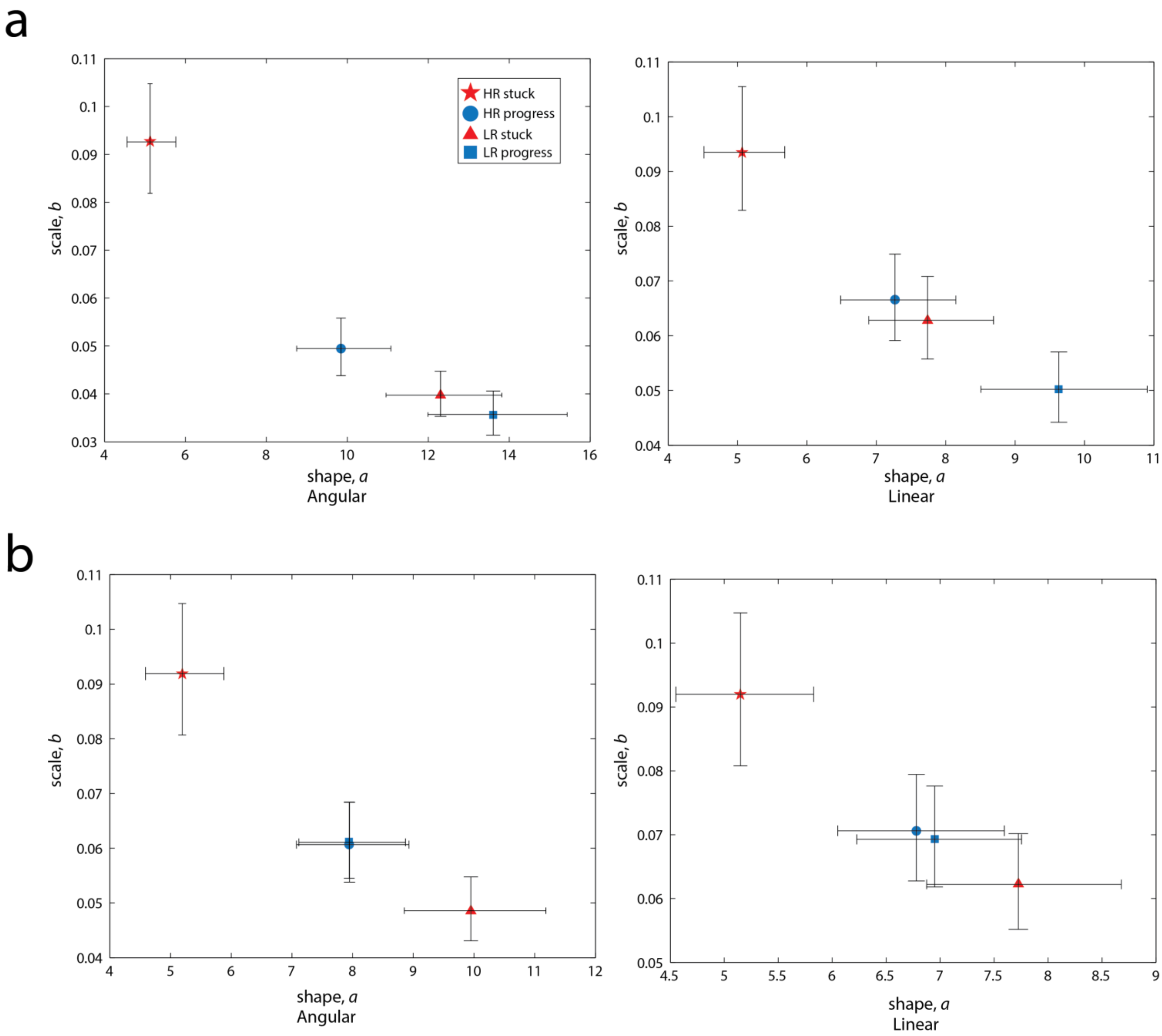

Supplementary Figure 7. Linking the rapidity of development with movement signatures for HR and LR participants: normalized peaks analysis. (a) shows data for 9-10 mo-olds and (b) shows data for 1-2 mo-olds. Parameter estimates are from normalized peaks of linear and angular speeds according to participants' developmental trajectories on the Mullen Early Learning Composite (ELC) scores. This figure uses same participant subgroupings as Figure 3 in the main text. Consistent with the outcome of the main analysis, HR infants with the most 'stuck' or delayed trajectory on the Mullen ELC show the most deleterious signatures (highest noise-to-signal levels, *b* parameter and lower shape, *a* parameter). Error bars denote 95% CIs.

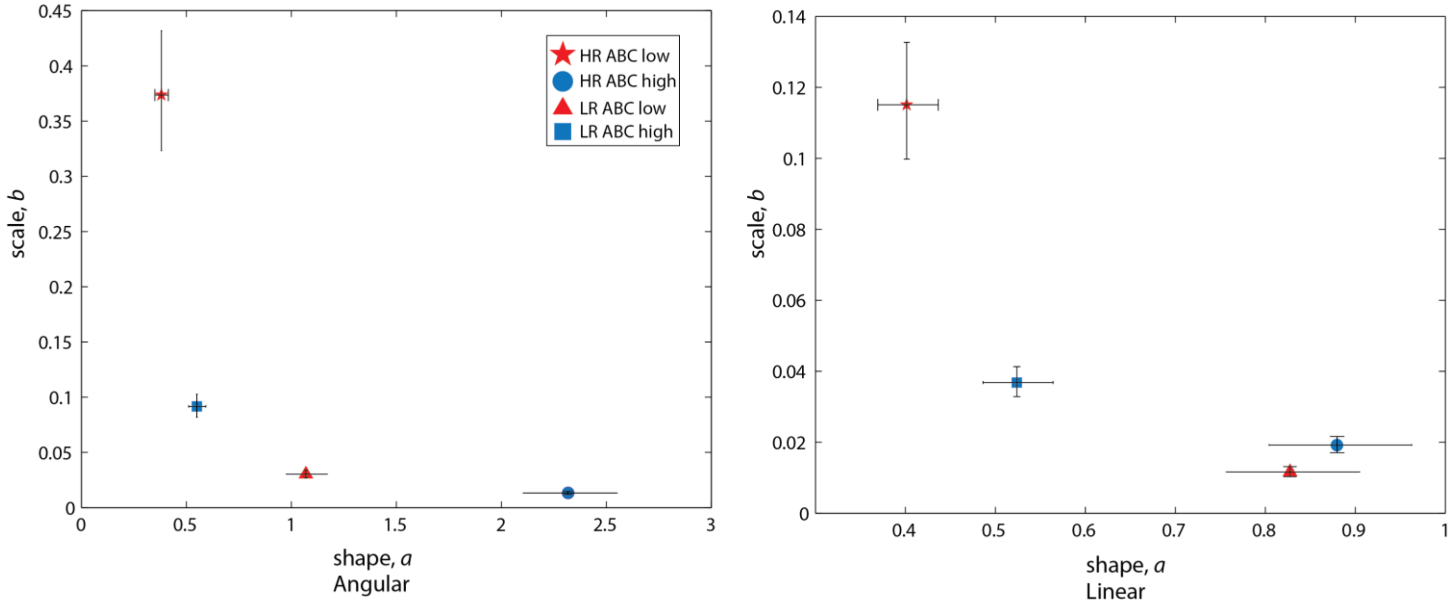

Supplementary Figure 8. Confirming the link between movement signatures and developmental outcomes for 9-10 mo-old HR infants using Vineland ABC scores obtained around 12 months. HR infants with the lowest ABC scores had the highest noise-to-signal levels and the least symmetric signatures relative to all other infants. Error bars denote 95% CIs. ABC: Adaptive Behavior Composite.

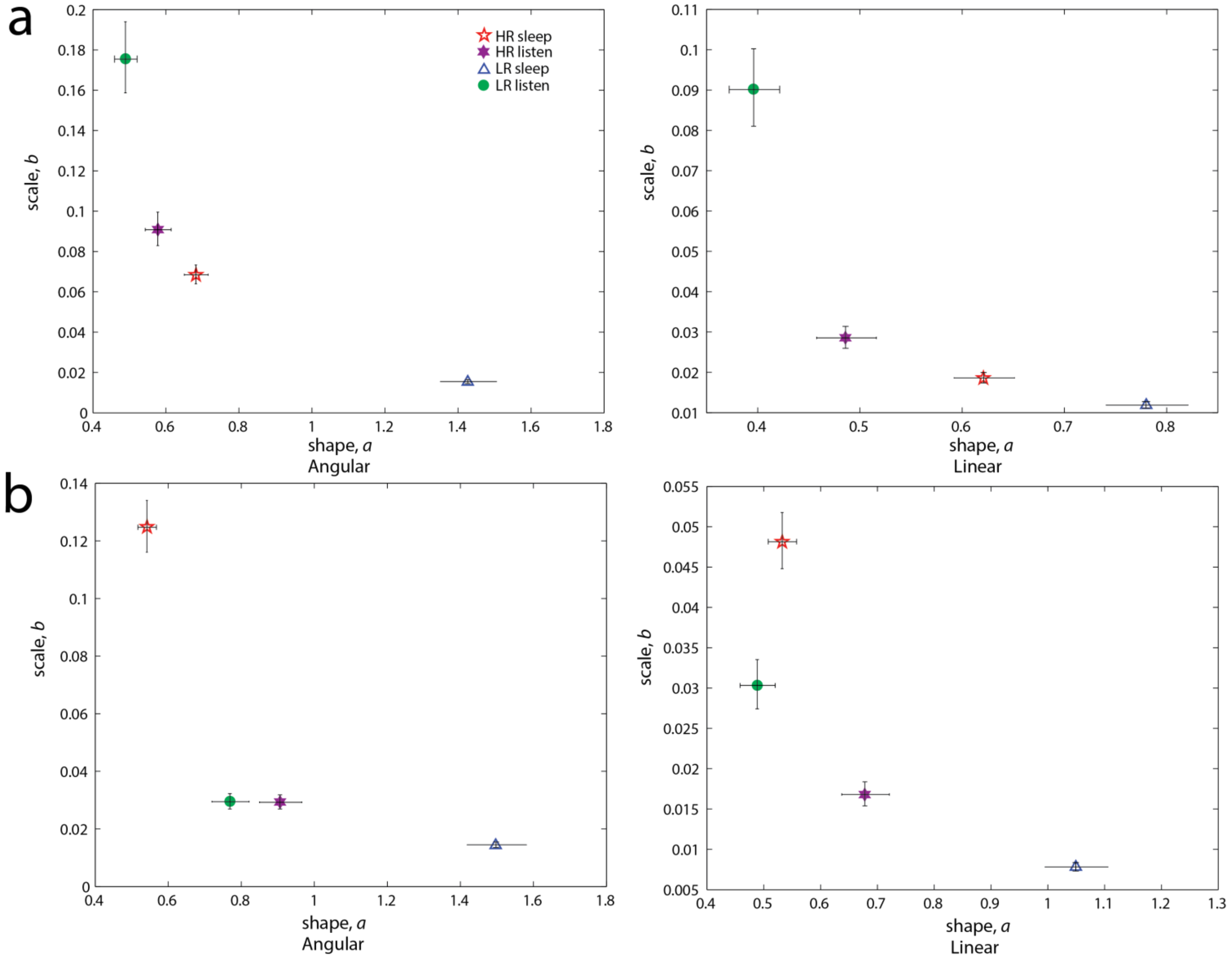

Supplementary Figure 9. Dissociation of movement signatures to "listen" vs. "sleep" conditions as a function of risk status and age: longitudinal subset. (a) Similar to patterns yielded by cross-sectional data, at 1-2 months, LR infants during sleep show the most symmetric signatures with high signal-to-noise levels and the noisiest, least symmetric signatures during wakefulness when listening to native language. In contrast, HR infants show more similar movement signatures during the two conditions. (b) At 9-10 months, HR infants show heightened noise levels during sleep and lower levels during the listening task relative to LR infants. *Note.* Longitudinal subset functional fMRI (native language listening task), total N=19: 1-2 mo-olds $N=10_{HR}$, $N=9_{LR}$, 9-10 mo-olds $N=10_{HR}$, $N=9_{LR}$. Longitudinal subset resting-state fMRI (sleep), total N=22: 1-2 mo-olds $N=11_{HR}$, $N=11_{LR}$, 9-10 mo-olds $N=11_{HR}$, $N=11_{LR}$. Error bars denote 95% CIs.

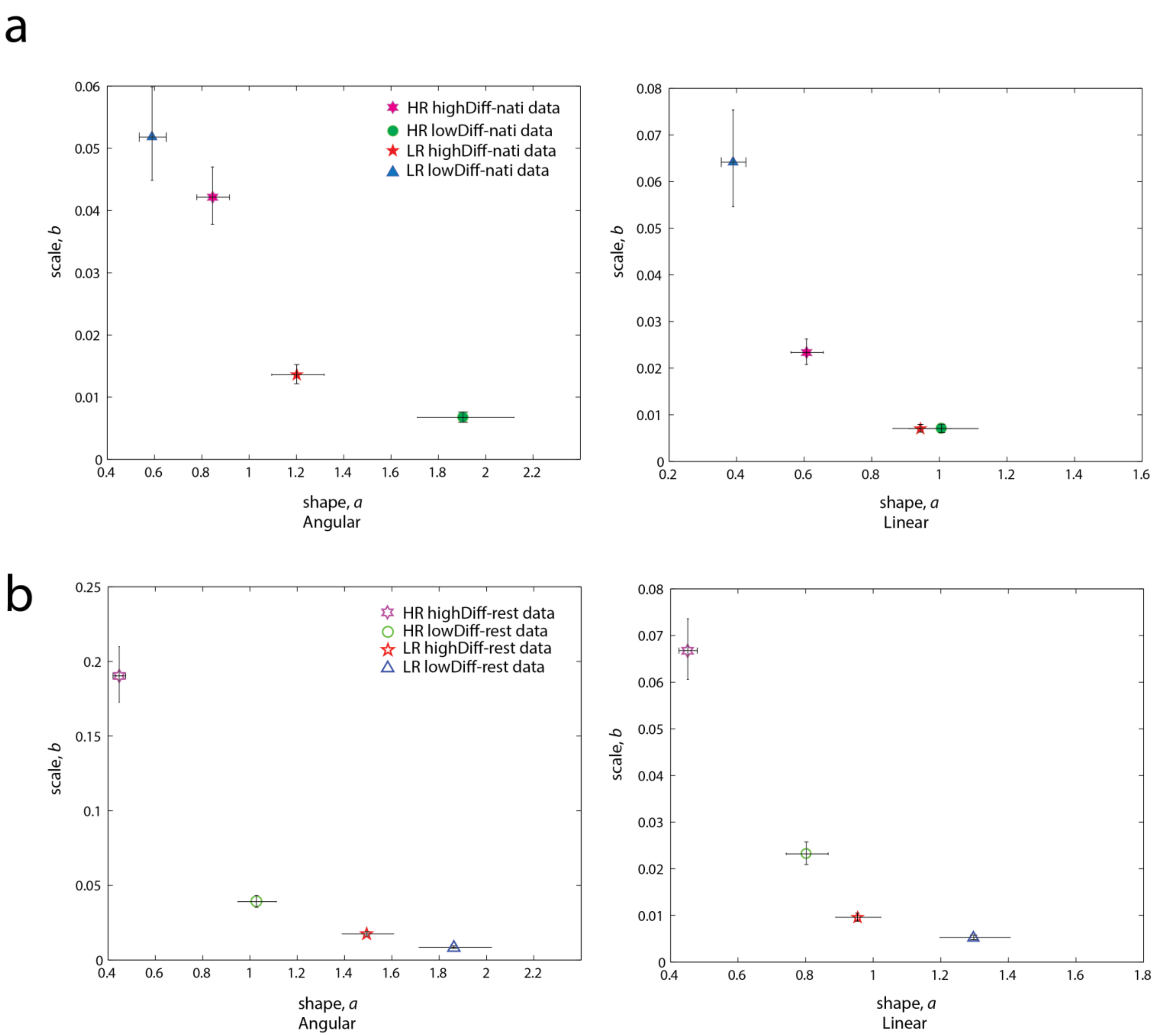

Supplementary Figure 10. Context-dependent movement signatures to "listen" vs. "sleep" conditions in 9-10 mo-olds as a function of FF difference in their movement signatures during the two scans as 1-2 mo-olds: longitudinal subset. (a), native language and (b), resting-state. *Note.* Longitudinal subset for infants with both functional fMRI native language listening scans and resting-state fMRI scans available at 1-2 months and 9-10 months, total N=19: $N=10_{HR}$, $N=9_{LR}$ (N=6 $HR_{highDiff}$, N=4 $HR_{lowDiff}$, and N=5 $LR_{highDiff}$, N=4 $LR_{lowDiff}$). "highDiff" indicates a positive difference between the *b* scale parameter (fano factor, FF) on the native scan minus the *b* scale parameter on the resting scan; "lowDiff" indicates a negative difference (native minus resting-state). Error bars denote 95% CIs.

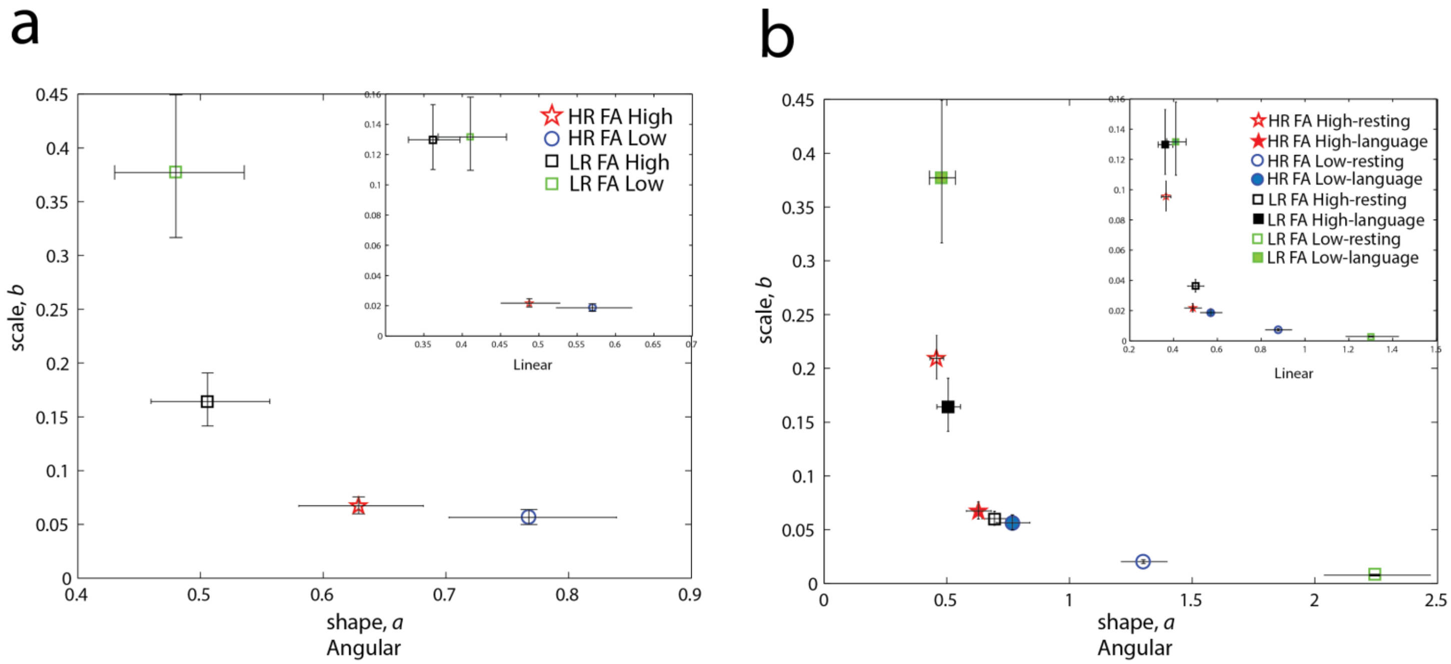

Supplementary Figure 11. Context-dependent movement signatures as a function of father's age. (a) native language scan signatures for 1-2 mo-old HR and LR infants, and (b), resting-state and native language scan signatures for 1-2 mo-old HR and LR infants. (a) shows that during the language listening task, LR infants with a younger father ("LR FA Low") have movement signatures characterized by heightened noise-to-signal levels that tend towards Exponential, less symmetric ranges, in contrast to LR infants with an older father who show the most normative movement signatures (non-overlapping 95% CIs on angular speed). HR infants with an older father ("HR FA High") have movement signatures characterized by heightened noise-to-signal levels that tend towards Exponential, less symmetric ranges, in contrast to HR infants with a younger father who show more normative movement signatures. Note that no matter how infants are grouped according to paternal age, during the language listening task, all LR infants had more noise-to-signal levels and signatures that were less symmetric relative to all HR infants. Examining (b), LR infants with the youngest fathers had the most diverse signatures (unfilled and filled green symbols). *Note*. For 1-2 mo-olds with available father's age data and both resting-state and native language scans: $HR_{FA\ High}$: N=6, $HR_{FA\ Low}$: N=5, $LR_{FA\ High}$: N=4, $LR_{FA\ Low}$: N=3, HR denotes "High Risk", LR denotes "Low Risk", FA denotes "Father's Age". Error bars denote 95% CIs.
